# Kinematics of Negative Headshake in Seven Sign Languages

**DOI:** 10.1162/OPMI.a.394

**Published:** 2026-10-03

**Authors:** Vadim Kimmelman, Anastasia Bauer, Carl Börstell, Jan Bulla, Allah Bux Sargano, Laurence Crettenand, Lorena Figueiredo, Anna Kuder, Hannah Lutzenberger, Dorcas Mihio, Marloes Oomen, Roland Pfau

**Affiliations:** Department of Linguistic, Literary and Aesthetic Studies, University of Bergen, Bergen, Norway; Department of Linguistics, University of Cologne, Cologne, Germany; Department of Mathematics, University of Bergen, Bergen, Norway; Deafness, Cognition and Language Research Centre, University College London, London, UK; Department of Linguistics, Stockholm University, Stockholm, Sweden; Department of Linguistics, University of Amsterdam, Amsterdam, The Netherlands

**Keywords:** headshake, negation, nonmanual markers, sign languages, kinematics, computer vision

## Abstract

In sign languages, negative sentences are often accompanied by nonmanual markers of negation, including headshake. In some sign languages, nonmanual markers alone can express negation, without the presence of a manual negative sign. While there has been considerable research on the typology of negation in sign languages in terms of syntactic structure and co-occurrence patterns of manual and nonmanual markers, very little research has been done on kinematic properties of negative headshake and their relation to linguistic features. In this exploratory study, we analyzed kinematics of negative headshake in seven sign languages—Brazilian Sign Language, Dutch Sign Language, German Sign Language, Kata Kolok, Norwegian Sign Language, Polish Sign Language and Russian Sign Language—using naturalistic corpus data (mainly free conversations). We used manual annotation of headshakes to analyze duration, and the computer vision tool 3DDFA_v2 to analyze amplitude and velocity. We considered whether headshake kinematics differs across languages, and whether it is affected by the presence of a manual negative sign and/or by spreading of the headshake across multiple signs. In addition, we compared three zones falling under the headshake: pre-negative sign, negative sign, and post-negative sign. We found that duration of headshakes varies cross-linguistically and is affected by spreading. For amplitude and maximum velocity, we found that spreading headshakes are kinematically stronger than non-spreading headshakes, but the presence of a manual negative sign does not significantly affect these kinematic measures. For zones within headshake, we found that, in general, the negative sign zone is kinematically weakest contrary to some previous findings for Dutch Sign Language, but that there is cross-linguistic variation in how the zones affect the kinematic measures. The study presents the first ever description of headshake kinematics in naturalistic data from several sign languages, and provides a first step towards typological research into kinematics of nonmanual markers.

## INTRODUCTION

Sign languages use nonmanual markers, such as facial expressions, mouth movements, head nods, headshake, and body leans in a wide variety of linguistic functions (Pfau & Quer, 2010). In negative sentences, several nonmanual markers are used to express negation, the most important of which is arguably the side-to-side headshake, which can be used to convey negation both with and without a co-occurring negative manual sign (Zeshan, 2004).

Consider example (1) from Brazilian Sign Language.^1^ In this example, negation is expressed by a combination of a manual negative sign never, and a headshake (hs) spreading over two of the signs. See also Figure 1, and a video recording of this example in the project repository.

(1) Brazilian Sign Language (Libras) (Quadros et al., 2020)
    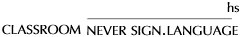    ‘Sign language was never used in the classroom.’ (F01, 00:12:25.454–00:12:28.582)

While there has been considerable research on the typology of negation in sign languages in terms of syntactic structure and co-occurrence patterns of manual and nonmanual markers (Makaroğlu, 2021; Oomen & Pfau, 2017), very little research has been done on the kinematic properties of negative headshake and their relation to linguistic features. We do not know whether headshake kinematics varies across sign languages, nor how it interacts with various grammatical properties of the negative constructions within languages, while we may expect there to be such relations, similar to how phonetics interacts with grammatical structure in spoken languages. However, technological advances and developments in creating sign language corpora (Fenlon & Hochgesang, 2022) have made it possible to attempt to answer such questions.

**Figure 1.**
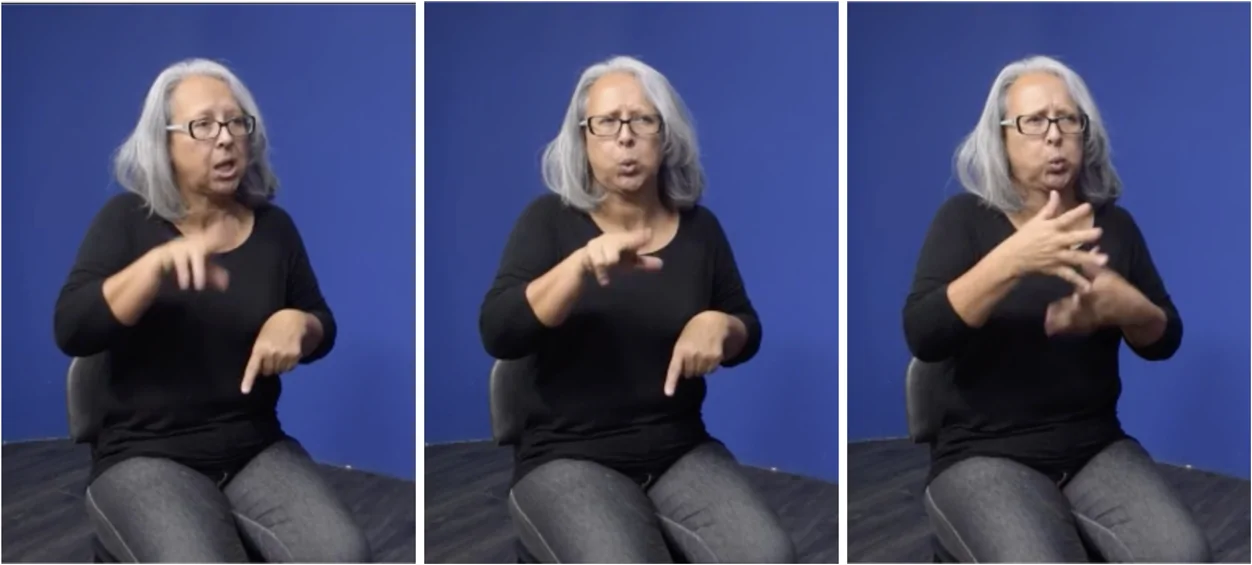
Snapshots from example (1) demonstrating a headshake. First two snapshots: sign never, last snapshot: sign sign.language.

On the technological side, computer vision has undergone rapid development in recent years, with several tools produced that allow automatic tracking of people’s movements in video recordings, including tracking facial landmarks, conducting 3D reconstruction, and calculating head rotation (e.g., Baltrusaitis et al., 2018; Lugaresi et al., 2019). In addition, many corpora of sign languages containing naturalistic data, such as free conversations, have been published in recent years—see Kopf et al. (2022) for an overview.

To date, only two small-scale studies have explored kinematics of negative headshake in naturalistic corpora of sign languages. Chizhikova and Kimmelman (2022) analyzed negative headshake in Russian Sign Language, and Kimmelman et al. (2024) in Dutch Sign Language. In this latter study, the authors did not just explore general kinematic properties of headshake, but also its internal kinematics, showing that headshakes can be divided into zones with different linguistic functions that are also kinematically different. However, these studies were based on very limited datasets, and no systematic cross-linguistic comparison of headshake kinematics has been done.

In the current exploratory study, we analyzed kinematics of negative headshake in seven sign languages: Kata Kolok, Brazilian Sign Language, Dutch Sign Language, German Sign Language, Norwegian Sign Language, Polish Sign Language and Russian Sign Language, using naturalistic corpus data. In total, we annotated 2,370 negative headshake instances across these languages. While these languages do not comprise a representative sample of sign languages of the world, their selection was motivated in part by (1) the availability of corpora and (2) the fact that they are geographically distributed across multiple continents (see Figure 2) and include both urban and rural sign languages,^2^ with very different estimated numbers of signers (see Table 1). Moreover, we know based on previous research that these sign languages present us with different typologies regarding the expression of negation, in particular, the interplay of manual and nonmanual markers of negation (see Negation in the Languages in This Study section for details). It seems reasonable to expect that these typological differences have repercussions on the kinematic properties of the headshake accompanying negative clauses. In this way, the present study makes an important contribution to sign language typology (that is, intra-modal typology; Zeshan & Palfreyman, 2017).

**Figure 2.**
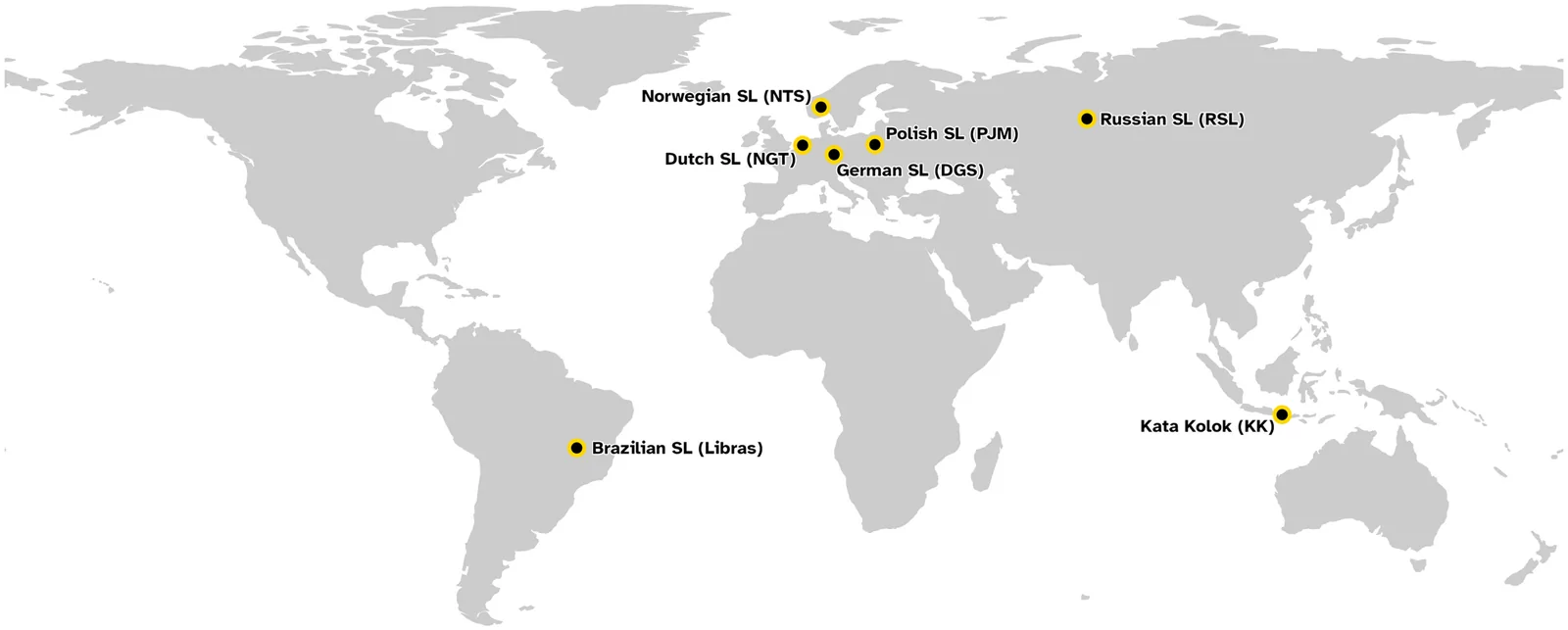
The geographical distribution of the languages of the study.

**Table 1.** Estimated number of signers per language.

**Language**	**Signers** (approximate)	**Source**
Brazilian Sign Language (Libras)	230,000–330,000 deaf, up to 630,000 total	Instituto Brasileiro de Geografia e Estatística (IBGE) (2020)
		Eberhard et al. (2022)
Dutch Sign Language (NGT)	10,000 deaf, up to 60,000 total	Klomp (2021, 55f)
German Sign Language (DGS)	80,000–200,000	Bross (2020a)
Kata Kolok (KK)	31 deaf, 1 500 total	de Vos (2012, 26), Lutzenberger (2022)
Norwegian Sign Language (NTS)	5,000 deaf, 16,500 total	Vonen (2020, 32)
Polish Sign Language (PJM)	9,000–50,000	2021 Polish National Census, Kalata-Zawłocka (2021)
Russian Sign Language (RSL)	120,000	Kimmelman (2014, 9)

We used the computer vision tool 3DDFA_v2 (Guo et al., 2018, 2020) to measure head rotation in order to quantitatively analyze headshake kinematics (amplitude and velocity) both within and across languages, focusing both on general kinematics and internal kinematics. The study is exploratory in nature, as we conduct a first ever exploration of headshake kinematics for most of the languages in our sample, and apply a large variety of new techniques and methods.

The paper is structured as follows. We first provide an overview of the properties of negative headshake in sign languages, including its use in each of the languages in our sample. We then discuss previous research on kinematics of nonmanual markers in sign languages and co-speech gestures. A separate section introduces the methodology, followed by a presentation of the results and a general discussion and conclusions. The data and scripts used in this paper can be accessed in this repository: https://doi.org/10.5281/zenodo.21979464.

## NEGATIVE HEADSHAKE IN SIGN LANGUAGES

### Negation and Negative Headshake

Negation is a domain of grammar that has been extensively studied for a considerable number of sign languages, including both urban sign languages from all continents as well as some rural sign languages (for recent overviews, see Gökgöz, 2021; Quer, 2020). Moreover, it is one of the grammatical domains that has given the impetus for sign language typology (Pfau & Zeshan, 2016; Zeshan, 2006a; Zeshan & Palfreyman, 2017), a research area within which the present study also situates itself, as we are going to compare patterns across seven sign languages. While the focus of our study will be on headshakes, it is important to start by providing some general information on nonmanual markers, specifically nonmanual markers of negation, and to introduce selected aspects of the grammatical encoding of negation across sign languages.

Nonmanual markers have been shown to fulfill functions at all levels of linguistic description in sign languages. For instance, nonmanual markers may differentiate minimal pairs in the lexicon (e.g., through mouthing^3^), contribute morphological information (e.g., through simultaneously realized facial expressions and mouth actions), and distinguish sentence types (e.g., through eyebrow and head movements)—see Pfau (2017) and Wilbur (2021). As for negation, the available studies show that all sign languages investigated to date employ a side-to-side headshake in the context of negation. However, next to headshake, other nonmanual markers have been attested in some sign languages, including a backward head tilt (e.g., in Turkish Sign Language, see Makaroğlu, 2021; Zeshan, 2006b), raised/lowered eyebrows (for Turkish Sign Language, see Gökgöz, 2011), negative facial expressions (e.g., in Chinese Sign Language; Yang & Fischer, 2002), and tongue protrusion (e.g., in Kata Kolok, a rural sign language of Bali; Lutzenberger et al., 2022). Clearly, at least some of these nonmanual markers are also commonly used by speakers of the surrounding hearing communities as gestural elements in the context of negation, and they are—to some extent—culture-specific (for the areal distribution of different types of head movements, see, e.g., Bross, 2020b; Jakobson, 1972).

However, within sign languages, it is not uncommon for (manual and nonmanual) gestural elements to lexicalize and grammaticalize (van Loon et al., 2014; Wilcox, 2004), and for the headshake in particular, it has been argued that it serves as a grammatical marker of negation in at least some sign languages (Pfau, 2015). Yet, this should not be taken to imply that headshake fulfills a grammatical function in all sign languages (see, e.g., Johnston, 2018, for Auslan, the majority sign language of Australia). In the present study, we scrutinized the use of headshake, as it (i) has been shown to function at the level of grammar and (ii) is commonly used in all sign languages in our sample (see next section for details). Headshake thus makes for an ideal candidate for a cross-linguistic phonetic comparison.

Not surprisingly, next to headshake, all sign languages studied so far also employ manual elements for encoding clausal and other types of negation: negative particles, negative indefinites (‘nothing, nobody’), negative adverbials (‘never’), negative modals (‘cannot, shouldn’t’), and other specialized manual negative signs, such as emphatic negatives and prohibitives. However, the ways in which manual and nonmanual markers interact at the clausal level is subject to interesting variation across sign languages (Zeshan, 2004, 2006a; also see Quer, 2020 for further discussion). As for manual markers, it has been shown that in some sign languages, the so-called *manual dominant* sign languages, the use of a manual negative sign is obligatory; a nonmanual marker may still be used (its use may even be common), but it cannot negate a clause by itself. Italian Sign Language (LIS) has been argued to belong to this group, which explains the contrast between (2a) and (2b). Whereas (2a) is a grammatical sentence, in which the manual negative sign usually occupies the clause-final position, (2b) is ungrammatical, independent of the scope of the headshake (“hs”), as it does not feature a manual negative sign.

(2) Italian Sign Language (LIS) (Geraci, 2006, 221)
  (a) 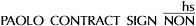       ‘Paolo didn’t sign the contract.’
  (b) 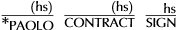       Intended: ‘Paolo didn’t sign the contract.’

In contrast, in *nonmanual dominant* sign languages, it is possible (and, in fact, common) to negate a clause by means of only a headshake, as is shown for American Sign Language (ASL) in (3). Yet, a manual negative sign may optionally be used, e.g., the preverbal negative particle not.

(3) American Sign Language (ASL) (Neidle et al., 1999, 45)
    
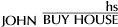
    ‘John is not buying a house.’

We observe that in both (2a) and (3), a headshake is used. However, the two types of sign languages have also been shown to differ with respect to properties of the headshake. In manual dominant sign languages, the scope of the headshake is usually confined to the manual negative sign—that is, it does not spread over adjacent signs, except for contexts in which the manual negative sign cliticizes to an adjacent sign (see, e.g., Wilkinson (2016) for reduction in negative collocations). In contrast, in nonmanual dominant sign languages, it is not uncommon for the headshake to spread over multiple signs of a clause—no matter whether a manual negative sign is present or not. For instance, in (3), the headshake spans over the entire verb phrase, which, according to Neidle et al. (1999), is obligatory in American Sign Language in the absence of the manual sign not. Further details regarding headshake spreading will be offered in the next section.

While the basic distinction between manual and nonmanual dominant sign languages still holds, recent studies have shown that also *hybrid* systems exist, which combine properties of both systems. In particular, there are sign languages in which the use of a manual negative sign is obligatory, but which still allow for the headshake to spread beyond the manual negative sign—e.g., Johnston (2018) for Australian Sign Language, Rudnev and Kuznetsova (2021) for Russian Sign Language, Pfau et al. (2022) for Georgian Sign Language, and Hodge et al. (2025) for British Sign Language. Indeed, Johnston (2018) shows that headshake spreading occurs frequently in Australian Sign Language, further noting that the domain of spreading does not seem to be constrained by syntactic structure in the language, as has been argued to be the case for some nonmanual dominant languages. Another hypothetical type of hybrid system would be one in which the use of a manual negative sign is optional, but without allowing for headshake spreading—e.g., the headshake might always accompany the main verb. To the best of our knowledge, such a sign language has never been described.

From a cross-modal perspective, nonmanual markers in general, and headshakes in particular, have been likened to tones in spoken languages. Just like tones, they (i) are suprasegmental, i.e., they associate with segmental slots in a simultaneous fashion, (ii) may fulfill lexical, morphological, and syntactic functions, and (iii) are capable of spreading (Pfau, 2017). As for point (ii), it is interesting to note that indeed all three functions have been assigned to headshake. Within and across sign languages, a headshake may be lexically specified (obligatory) for manual negative signs, it may function as a simultaneous affix that attaches to a verb, and, in certain syntactic theories, it has been argued to spell out a syntactic feature hosted by a dedicated syntactic head.

### Negation in the Languages in This Study

Having addressed some typological aspects regarding the use and interplay of manual and nonmanual markers of negation, we now briefly describe the form and structure of negation in the seven sign languages included in this study, in alphabetical order. For all languages, we exemplify common patterns, using examples that are also part of the dataset used for the present study.

#### Brazilian Sign Language (Libras).

Brazilian Sign Language (Libras; *Língua Brasileira de Sinais*) has been characterized in the literature as a nonmanual dominant language. Arrotéia (2005) was the first author to present a detailed description of negation in Brazilian Sign Language. Based on elicited data, she identified facial negation—eyebrow lowering, lip corners down or an O-shaped mouth, and a head nod—as the obligatory marker of negation (Arrotéia, 2005). She also analyzed the use of headshakes and negative signs separately, but argued that the former function primarily as affective markers and that negative clauses marked only by headshakes are considered ungrammatical. More recently, a corpus-based study of negation in Brazilian Sign Language (Garcia, 2024) supported Arrotéia’s (2005) findings—particularly the characterization of Brazilian Sign Language as a nonmanual dominant language, focusing on the role of mouth articulation.

In the data analyzed for the present study, although nonmanual marking is highly frequent, negative manual signs occur even more frequently. These findings challenge the characterization of Brazilian Sign Language as a language with nonmanual dominant negation; rather, we consider it a hybrid system.

Headshakes tend to co-occur with the manual negative sign, and may spread—as in example (4a)—or be restricted in scope of the negative sign—as illustrated in (4b).

(4) Brazilian Sign Language (Libras) (Quadros et al., 2020)
  (a) 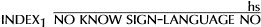       ‘I didn’t know sign language’ (M09, 00:00:33.395–00:00:34.436)
  (b) 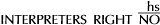       ‘There was no right to interpreters’ (F09, 00:10:23.790–00:10:25.481)

#### Dutch Sign Language (NGT).

In her dissertation, Coerts (1992) investigated the use of various grammatical nonmanual markers in Dutch Sign Language (NGT; *Nederlandse Gebarentaal*), including negative nonmanuals, making Dutch Sign Language one of the first sign languages for which negation has been described in detail. Coerts’ findings, which are based on data elicited from 16 signers, suggest that Dutch Sign Language can be classified as a nonmanual dominant sign language, as 62% of negative clauses in her dataset are negated by means of a nonmanual marker only. 25 years later, Oomen and Pfau (2017) studied negation based on naturalistic data from the Corpus NGT (Crasborn et al., 2008) and came to the same conclusion. In their dataset—which, in contrast to Coerts’ data, excludes clauses with neg-words (e.g., nothing, never), negative modals, and the negative completive not.yet—nonmanual-only negation is found in 58% of the clauses.

Oomen and Pfau (2017) add a detailed analysis of the scope of headshake and observe that the headshake (i) always accompanies the manual negative sign (when present), (ii) accompanies the predicate in 95% of the examples, (iii) may optionally spread onto the object argument, (iv) may spread onto the subject (if pronominal), and (v) may spread onto prosodically light clause-final signs (e.g., pronouns). Two representative examples are provided in (5a)–(5b). In (5a), the headshake accompanies the clause-final negative particle as well as the verb preceding it; in (5b), the headshake accompanies the verb phrase as well as the clause-final pronominal pointing sign index_3_.

(5) Dutch Sign Language (NGT) (Oomen & Pfau, 2017, 25, 27)
  (a) 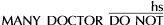       ‘Many doctors don’t do that.’ (531-S025-01:55)
  (b) 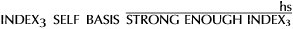       ‘Their basis isn’t strong enough.’ (386-S019-00:22)

In line with what had previously been suggested for German Sign Language (see below), Oomen and Pfau (2017) conclude that the headshake accompanying not is lexically specified, while the headshake coarticulated with the predicate is morphological (i.e., affixal) in nature. This implies that we are actually dealing with two distinct headshakes in (5a)—however, phonetically, these are fused into a single contour. The authors further assume that headshake accompanying other clausal elements, such as pronouns, results from prosodic spreading (for an overview of Dutch Sign Langauge negation, see also Klomp et al., 2025).

#### German Sign Language (DGS).

Clausal negation in German Sign Language (DGS; *Deutsche Gebärdensprache*) has been studied in a series of works by Pfau (2002, 2008, 2016). Beyond describing the patterns of negation, Pfau (2015) also offers a formal syntactic analysis as well as speculations about the grammaticalization of negation. Note, however, that these investigations are based on elicited data and grammaticality judgments, not on corpus data. Data from the DGS Korpus (Konrad et al., 2020) support Pfau’s conclusion that German Sign Language displays a nonmanual dominant negation system. Based on the observation that the headshake is always minimally associated with the predicate—no matter whether a manual negative sign is present or not—Pfau argues that the headshake functions as a (featural) affix that attaches to the verb. He further proposes that German Sign Language presents us with a system of split negation, where one of the two elements, the manual particle, is optional (not unlike Colloquial French: *je (ne) sais pas* ‘I don’t know’, expressed as ‘I (neg) know neg).

Two representative examples from the DGS Korpus (Konrad et al., 2020) are presented in (6a) and (6b). Example (6a) features the manual negative sign not in post-predicate position (a common position for the negative particle) and a headshake accompanying the adjectival predicate afraid, the negative particle, and the clause-final pronoun copy. For an example like this, Pfau (2016) would argue that the headshake on not is lexical, the headshake on the predicate morphological, and that the one accompanying the pronoun copy results from prosodic spreading, akin to spreading of tone (*tone sandhi*) in spoken languages. In contrast, (6b) is negated by headshake only, which in this case spans over the verb phrase.

(6) German Sign Language (DGS) (Konrad et al., 2020)
  (a) 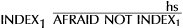       ‘I was not afraid.’ (1414563, 09:12)
  (b) 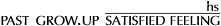       ‘While growing up, I was not satisfied.’ (1247835, 01:23)

#### Kata Kolok (KK).

Kata Kolok marks negation using a manual particle neg and a headshake. Generally, neg occurs in postpredicative or clause-final position. The negation system in Kata Kolok has initially been described as displaying a strongly manual dominant pattern in which the manual particle neg is used obligatorily and the headshake is small and optional (Marsaja, 2008). Lutzenberger et al. (2022) used conversational data from signers from three subsequent generations to revisit this claim, and found a much more variable pattern in which both the manual particle and the headshake are frequently used. While the data include some examples in which no headshake is used at all (see (7a)), the most common strategy among Kata Kolok signers is one that combines the manual negative sign neg and a headshake (see (7b)).

(7) Kata Kolok (KK) (Lutzenberger et al., 2022, 10–11)
  (a) index_1_ drink sweet neg
       ‘She does not drink tea.’
  (b) 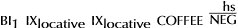       ‘I don’t take my coffee over here.’

Since Lutzenberger et al.’s (2022) dataset includes instances of manual-only as well as nonmanual-only negation—i.e., utterances in which exclusively a manual negative sign or a negative headshake are used to mark negation—they suggest that there is no clear evidence for one specific negation system in Kata Kolok. Nevertheless, in line with Marsaja’s (2008) observation, the headshake most commonly coincides with only the manual negative sign, while spreading of the headshake over multiple signs occurs only occasionally (see (8)). Beyond these observations, Lutzenberger et al. (2022) also report that another nonmanual marker, namely tongue protrusion, is commonly used to mark negative existentials.

(8) Kata Kolok (KK) (Lutzenberger et al., 2022, 12)
    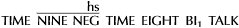    ‘I was told to come at eight o’clock in the morning, not at nine.’

#### Norwegian Sign Language (NTS).

Negation in Norwegian Sign Language (NTS; *Norsk tegnspråk*) has not been described before in any detail. Vonen (2020, 67) argues that negation realized via headshake only without a manual negative sign is possible, but does not provide examples illustrating it.^4^ Vonen (2020, 64) offers example (9), which includes both the manual negative adverb never and a headshake.

(9) Norwegian Sign Language (NTS) (Vonen, 2020, 64)
    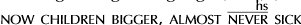    ‘Now that the children are bigger, they are almost never sick.’

In our dataset, which is part of Ferrara and Ringsø (2021), we found examples of negative sentences both with and without a manual negative sign. (10a) shows an example with the general manual negative sign not and headshake spreading, while (10b) shows an example without a manual negative sign and with headshake spreading.

(10) Norwegian Sign Language (NTS) (Ferrara & Ringsø, 2021)
  (a) 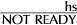       ‘[I was] not ready.’ (DPNTS_B_MS_A, 00:25:59)
  (b) 
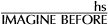
       ‘[Have you] not imagined it before?’ (DPNTS_B_MS_A, 00:13:52)

#### Polish Sign Language (PJM).

Negation in Polish Sign Language (PJM; *Polski język migowy*) has been investigated primarily in corpus-based studies by Kuder et al. (2018) and Kuder (2021), which reveal a highly variable system involving both manual and nonmanual strategies. Polish Sign Language employs all major negative sign types, including a general negative particle (glossed as no), morphologically negated verbs (formed by means of a bound negative prefix realised as a circular wrist movement articulated immediately before the target sign, cf. Tomaszewski (2015)), negative modals, negative possessives, and negative words such as never and nothing. Among these, verbs negated morphologically as well as verbs exhibiting suppletive negative forms constitute the most frequent strategies for clausal negation in the corpus data.

Headshake is a primary nonmanual negative marker in Polish Sign Language (Kuder, 2021). While headshake commonly co-occurs with manual negation (11a), it is not obligatory: manual negative signs occur with and without headshake in roughly equal proportions. Conversely, clauses negated exclusively by headshake are attested, but they are relatively rare and account for only a small fraction of negative clauses (Kuder, 2021). This distribution makes it difficult to classify Polish Sign Language straightforwardly as either manual dominant or nonmanual dominant.

With respect to scope and spreading of headshake, Polish Sign Language displays a high degree of variation. When present, headshake most often accompanies only the negated sign and only rarely spreads to other clause constituents. Importantly, in clauses negated exclusively by headshake, spreading is virtually absent, with the nonmanual typically constrained to the negated element (11b), contrary to expectations for nonmanual dominant languages in Zeshan’s typology. No systematic correlation between headshake spreading and syntactic structure has been identified, and many headshakes occurring in negative environments serve pragmatic or discourse-related functions rather than encoding grammatical negation (Kuder, 2021).

Two representative corpus examples are given in (11a)–(11b). In (11a), negation is expressed morphologically on the verb and accompanied by a spreading headshake; in (11b), the clause is negated solely by headshake, without any manual negative marker.

(11) Polish Sign Language (PJM) (Kuder, 2020, 190; 167)
  (a) 
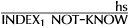
       ‘I don’t know.’
  (b) 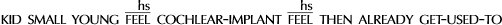       ‘Small children do not feel the cochlear implant; only later do they get used to it.’

#### Russian Sign Language (RSL).

According to published research on Russian Sign Language, a manual negative sign is required to express negation (Chizhikova & Kimmelman, 2022; Rudnev & Kuznetsova, 2021)—compare examples (12a) and (12b). The manual negative sign typically occurs in the post-verbal, clause-final position (Rudnev & Kuznetsova, 2021). Negative nonmanual markers, which include headshake, can accompany the manual negative sign, although their use is highly optional (see Chizhikova & Kimmelman, 2022). Although infrequent, the headshake can also spread beyond the manual negative sign, as in (12a), suggesting that Russian Sign Language features a hybrid system.

(12) Russian Sign Language (RSL) (Rudnev & Kuznetsova, 2021, 260ff)
  (a) 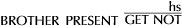       ‘My brother didn’t get a present.’
  (b) 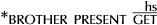       Intended: ‘My brother didn’t get a present.’

In the data used for the current study, we also found a pattern where the negative sign itself is not accompanied by a headshake, but a headshake spreads over other signs, which, to our knowledge, has not been previously described for Russian Sign Language—see (13).

(13) Russian Sign Language (RSL) (Bauer & Poryadin, 2023)
    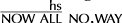    ‘Now there is no way.’

#### Summary.

In all the languages in our sample, negation is typically expressed by a combination of manual and nonmanual means, with headshake being an important marker. However, the specific patterns of headshake use differ between languages. In terms of manual dominance, Dutch Sign Language, German Sign Language, and possibly Norwegian Sign Language can be characterized as nonmanual dominant, as they frequently employ headshake alone to express negation, and headshake often spreads. Kata Kolok, Brazilian Sign Language, Polish Sign Language, and Russian Sign Language are hybrid systems, but this constitutes a broad category, see also Table 2. In all of the hybrid languages, there is—to various degrees—a preference for having a manual negative sign in negative sentences, but some purely nonmanual instances of negation are also reported. Headshake spreading is also possible in all of the hybrid languages, but with varying frequencies and scopes. Among the hybrid languages, Kata Kolok and Russian Sign Language show most manual dominance in terms of frequency of the presence of the manual negative marker, so we use the label hybrid/manual for them.

**Table 2.** Number of signers, age group and typological classification of the languages.

**Language**	**Age group (years)**		**Total number of signers**	**Dominance**
	≤45	>45		
German Sign Language (DGS)	37	28	65	nonmanual
Dutch Sign Language (NGT)	22	0	22	nonmanual
Norwegian Sign Language (NTS)	15	3	18	nonmanual
Polish Sign Language (PJM)	14	4	18	hybrid
Brazilian Sign Language (Libras)	6	4	10	hybrid
Russian Sign Language (RSL)	5	2	7	manual/hybrid
Kata Kolok (KK)	4	2	6	manual/hybrid

Taken together, these patterns suggest that the classification of sign languages presents us with a continuum from more to less nonmanual dominant and less to more manual dominant, where none of the sign languages fully aligns with the criteria previously proposed for manual dominant sign languages in Zeshan (2004). Based on the descriptions above, we may categorize Dutch Sign Language, German Sign Language, and Norwegian Sign Language as leaning more toward the nonmanual dominant end of the continuum, while Russian Sign Language, Polish Sign Language, Brazilian Sign Language, and Kata Kolok can be positioned more toward the manual dominant end, although they might be better classified as hybrid languages. We hypothesize that the kinematic properties of headshake relate to the positioning of a sign language on this continuum, where we expect headshakes to generally be kinematically stronger in more nonmanual dominant sign languages, for which the headshake fulfills a more pronounced grammatical function. We outline our specific hypotheses further below.

## KINEMATICS OF NONMANUAL MARKERS

Kinematic characteristics of nonmanual markers in sign languages as well as of nonmanual co-speech gestures have been vastly under-researched in comparison to phonetics and intonation of spoken languages, due to both a limited research focus on the visual modality of natural languages, and the technical challenges of studying the visual modality. However, some studies focusing on nonmanual markers including headshake exist, and in recent years, more studies are appearing that use computer vision to explore the kinematics of nonmanuals in sign languages and co-speech gesture.

### Motion Tracking Studies

Some studies have looked at head motion accompanying speech in spoken languages, also studying its kinematics with the help of tools such as motion capture (e.g., Hadar et al., 1983, 1985), or with electromagnetic articulography (e.g., Frid et al., 2018, 2019). Head movement is typically described as involving rotations along three axes: *pitch* (corresponding to nodding up and down), *yaw* (corresponding to headshakes and turns side to side), and *roll* (corresponding to sideward tilts of the head). In addition, the head can remain fixed with regard to the rotational axes, but move in three-dimensional (3D) space: left/right, up/down, and front/back. It has been found that different functions of head movements and different contexts in which these movements are used, can be kinematically different in terms of velocity, amplitude, etc. (for an overview, see Wagner et al., 2014), and that head movements (and other nonmanual co-speech gestures) are often coordinated with speech and aligned with prominence peaks in prosody (Ambrazaitis & House, 2023); see also Momsen and Coulson (2025) for a more general study of the relation between speech and gesture kinematics.

For sign languages, we have not found kinematic studies of negative headshake using motion capture, but Puupponen et al. (2015) analyzed kinematics of head nods, thrusts and pulls in a small motion capture dataset of Finnish Sign Language. They report on measures of duration and amplitude of head motion in relation to different types and functions of head movements.

### Computer Vision-Based Studies

Following the successful application of deep learning approaches to computer vision, several toolkits for detecting and tracking body landmarks in video recordings have appeared in recent years, including OpenPose (Cao et al., 2018) and MediaPipe (Lugaresi et al., 2019). Some of these tools, such as OpenFace (Baltrusaitis et al., 2018) and 3DDFA_v2 (Guo et al., 2018, 2020), also feature automatic 3D reconstruction from 2D video recordings as well as head rotation tracking, which is the very reason we used the latter tool in the current study.

The earliest studies using computer vision tools to analyze sign languages have appeared over a decade ago (Karppa et al., 2014; Metaxas et al., 2012). In recent years, a large number of publications applying new computer vision models to sign language and gesture data has appeared (see, for example, Börstell, 2023; Börstell & Lepic, 2020; Östling et al., 2018; Trujillo et al., 2019), also for analyzing nonmanual markers (Chizhikova & Kimmelman, 2022; Kimmelman et al., 2020), including head nods (Bauer et al., 2024; Karabüklü et al., 2026) and headshake (Chizhikova & Kimmelman, 2022; Kimmelman et al., 2024).

For headshake specifically, Chizhikova and Kimmelman (2022) have previously analyzed negative headshake kinematics in Russian Sign Language using the yaw rotation measurements from OpenFace for a small number of headshakes found in the online corpus of Russian Sign Language (Burkova, 2015). They explored the kinematic characteristics of amplitude and the number and frequency of peaks (reflecting the number of rotational head turns), but failed to find evidence that these characteristics correlate with the specific manual negative sign used. Kimmelman et al. (2024) applied the same methodology to analyze negative headshakes occurring in Corpus NGT (Crasborn et al., 2008). They found that spreading headshakes, unsurprisingly, are longer in duration, but not different in amplitude or velocity from non-spreading headshakes. In addition, they found that the headshakes have higher velocity and amplitude over the manual negative signs and verbs—that is, when they have a lexical or morphological function, see Oomen and Pfau (2017)—in comparison to when they spread over other signs (i.e., a prosodic function). At the same time, they also observed an overall prosodic weaking of the headshake with time: it tended to be kinematically stronger in the beginning and weaker by the end, in parallel to downstep or declination of pitch in spoken languages (Pierrehumbert, 1980). The current study can be seen as an extension of this approach to a much larger cross-linguistic dataset.

## HYPOTHESES

In this study, we primarily explored the effects of spreading and the presence of a manual negative sign on kinematics of headshakes (*general kinematics*), and on kinematic differences between different zones of a headshake (*internal kinematics*). For both types of analysis, we formulate general hypotheses as well as hypotheses that relate to the typological classification of the seven languages in our sample presented earlier. We also include age as a possible predictor of headshake kinematics, as, in general, motion of body parts can be affected by age (Doriot & Wang, 2006). It is important to note that, given the novelty of the method and the large number of potential measures and factors that can affect outcomes, the study should be seen as exploratory.

### General Headshake Kinematics

1. We hypothesize that languages will vary in headshake kinematics, and that this variation will be related to the typological profile of the languages. Specifically, we hypothesize stronger headshake kinematics in the sign languages toward the nonmanual dominant end of the continuum (German Sign Language, Dutch Sign Language, and Norwegian Sign Language), given that the headshake is the primary marker of negation.
2. We hypothesize that spreading headshakes are longer than non-spreading headshakes (i.e., have longer duration), and that headshakes co-occurring with a manual negative sign are longer because they potentially overlap with more signs.
3. We hypothesize that spreading headshakes are kinematically weaker (i.e., have lower amplitude and median and maximum velocity) than non-spreading headshakes due to their hypothesized longer duration, making them more prominent without the need for stronger kinematic features. We thus expect a compensatory relation between headshake spreading and duration on the one hand, and other kinematics on the other hand.
4. We hypothesize that the presence of a manual negative sign is negatively correlated with the kinematic measures, due to the headshake not being the sole expression of negation in the sentence and thus lessening the importance of visual and kinematic prominence. We thus expect a compensatory relation between the presence of a manual negative sign and kinematics.
  - We hypothesize that the effect is relatively strong in sign languages toward the manual dominant end of the continuum (Russian Sign Language, Polish Sign Language, Brazilian Sign Language, and Kata Kolok), since in these languages, manual and nonmanual negative elements contribute comparably to the expression of negation. Thus, when a manual negator is present, there is less of a need for the headshake to be visually and kinematically prominent.
  - We hypothesize that the effect is relatively weak in sign languages that are nonmanual dominant (Dutch Sign Language, German Sign Language, and Norwegian Sign Language) given that in these languages, the nonmanual marker is the primary marker of negation while the manual sign is less influential. As such, we expect the headshake to be generally quite prominent visually and kinematically, independent of whether or not a manual negator is present as well.

### Internal Headshake Kinematics

For internal headshake kinematics, we have two alternative hypotheses, following the findings in Kimmelman et al. (2024).

If headshake is intrinsically linked to the manual negative sign (i.e., is a phonological component of a manual negative sign), as is generally assumed, we predict it to be kinematically stronger (i.e., have higher amplitude and velocity measures) over the manual negative sign itself, and weaker in the other zones. We expect this to be the case for all seven sign languages, independent of their typological classification, because we assume that headshakes accompanying manual negative signs are always lexically specified. Thus, for all sign languages, we expect the headshake to be kinematically stronger over the manual negative sign because there it serves a more prominent grammatical function than elsewhere in the sentence, where it—mostly—fulfills a prosodic function.^5^

If headshake follows a stable prosodic contour, independent of the manual signs it accompanies, we expect it to be kinematically stronger in the beginning and weaker by the end, similar to pitch downstep in spoken languages.

To test these hypotheses, we analyze the kinematic properties in three different zones: before (labeled “pre”), on (“NEG”), and after (“post”) the manual negative sign. The predictions under the two hypotheses are schematized in Figure 3.

**Figure 3.**
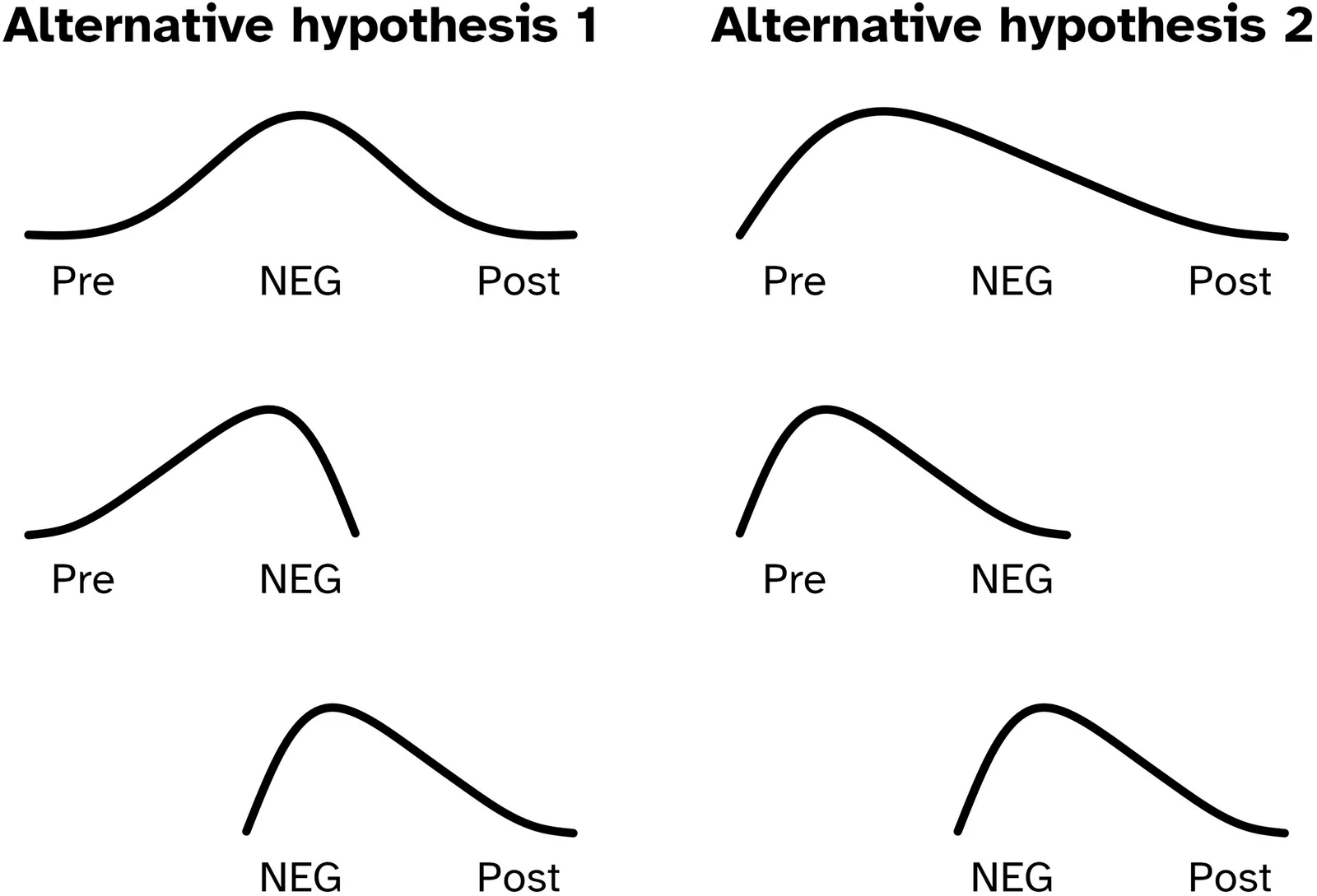
Visualization of internal headshake kinematic dynamics according to the two hypotheses: alternative hypothesis 1 posits that headshake amplitude and velocity should be the highest when coinciding with the negative sign (“NEG”); alternative hypothesis 2 posits that headshake amplitude and velocity should be the highest at the beginning of the headshake and then decrease.

## METHODS

### The Dataset

The dataset contains 2,370 instances of headshake, which are not equally distributed between the languages (see Figure 4). German Sign Language constitutes the largest subset, containing 703 headshakes, while Kata Kolok and Dutch Sign Language are the smallest ones, with 224 and 210 headshakes, respectively.

**Figure 4.**
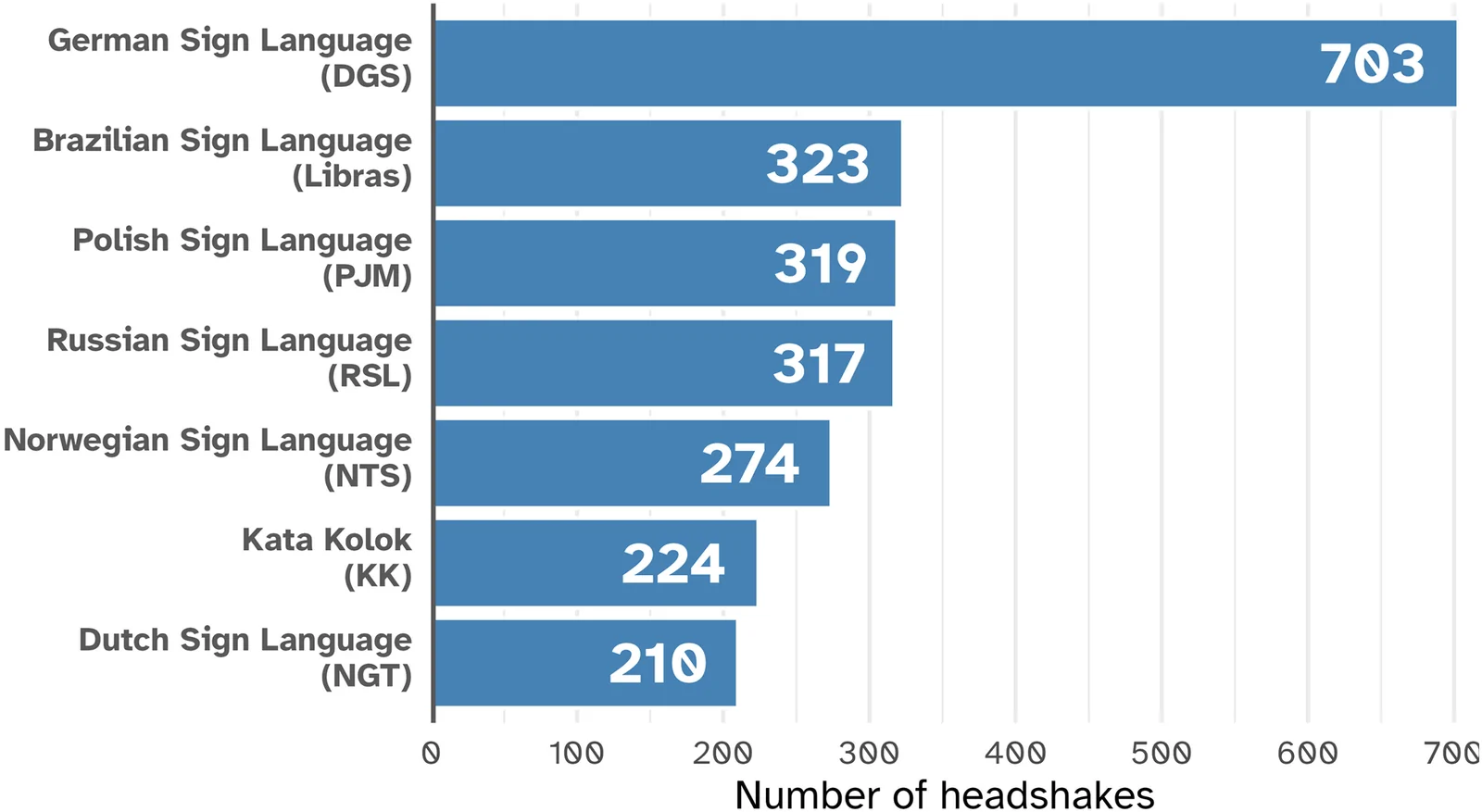
Number of headshake instances per language in the dataset.

The data come from 146 signers in total, but the number of signers is also unevenly distributed across the individual languages, ranging from 6 for Kata Kolok and 7 for Russian Sign Language, to 65 for German Sign Language (see Table 2).^6^ For all languages, ELAN (Crasborn & Sloetjes, 2008) annotation files with (at least) sign glosses and translations were available. Importantly, the data analyzed in this study derive from semi-natural, dyadic^7^ interactions. This design enhances ecological validity and facilitates cross-linguistic comparability.

The Brazilian Sign Language data were extracted from the *Deaf of Reference* section of the *Corpus de Libras* (Quadros et al., 2020), which includes 36 interviews with researchers, professors, and deaf individuals involved in deaf movements across different Brazilian states. For this study, we selected data from 10 signers aged between 29 and 61 years (*M* = 42.5, *SD* = 9.5).

The Dutch Sign Language data come from the Corpus NGT (Crasborn et al., 2008). The public part of the corpus contains 2.280 recordings with 100 adult Dutch Sign Language signers from various regions in the Netherlands. For this study, we used the same set of 35 annotated video files as Oomen and Pfau (2017) used in their study. The recordings include 22 signers, all from the Groningen region in the north of the Netherlands. The signers interact in dyads, discussing a variety of topics related to sign language and deaf culture. At the time of recording, 2 signers were younger than 20, 10 belonged to the 21–30 age group, 4 to the 31–40 age group, and 6 to the 41–50 age group.

The German Sign Language data come from the *MY DGS annotated Public Corpus of German Sign Language* (Konrad et al., 2020). The corpus contains annotated video recordings of 328 signers from different regions of Germany, engaging in a variety of linguistic activities. For the current study, we selected free conversations and discussions of experiences of deaf individuals, produced by 65 signers. Of these signers, 12 belong to the 18–30 age group, 25 to the 31–45 age group, 17 to the 46–60 age group, and 11 to the over 61 age group.

The Kata Kolok data stem from the *Kata Kolok corpus* (de Vos et al., 2007). This dataset comprises naturalistic and elicited data from Kata Kolok signers from generation three through five. For this study, three spontaneous, dyadic conversations have been selected, featuring a total of six deaf signers (the same dataset was used in Lutzenberger et al., 2022). Dyads are matched for gender and age (generation), representing generations three (male, aged around 60s), four (female, aged around mid-30s–40s), and five (male, aged around late teens/early 20s), but the conversations vary in length.

The Norwegian Sign Language data come from the dataset *Norwegian Sign Language corpus: Depicting Perspective* (Ferrara & Ringsø, 2021). This dataset contains conversations between pairs (and, occasionally, larger number) of signers from Bergen, Oslo and Trondheim, focused on spatial language—see Ferrara and Nilsson (2017), Ferrara and Ringsø (2019), and Ferrara (2019) for research using this dataset. For this study, we selected recordings by 18 signers. 10 of the signers belong to the 18–29 age group, 5 signers to the 30–45 age group, 3 signers to the 45–60 age group.

The Russian Sign Language dataset is drawn from a recently established and still developing resource, the *Corpus of RSL Conversations* (Bauer & Poryadin, 2023). The corpus consists of video recordings of casual dyadic conversations in Russian Sign Language, each lasting between 40 and 60 min. The participants are deaf native signers of Russian Sign Language currently residing in Germany. At present, the corpus includes recordings of seven deaf native Russian Sign Language signers (*M* = 46.3, *SD* = 25–75) who previously lived in Russia prior to immigrating to Germany.^8^ Most participants were well acquainted with one another. Their conversations covered a range of topics, including their lives in Russia before immigration and their experiences as deaf individuals in both Europe and Russia.

The Polish Sign Language data were extracted from the *Polish Sign Language Corpus* compiled at the University of Warsaw by the Section for Sign Linguistics (Rutkowski et al., 2017). The corpus consists of high-definition video recordings of 150 deaf signers interacting in dyads under controlled studio conditions and follows a standardized elicitation protocol combining free conversation (which we analyze in the current study), narrative tasks, and semi-structured elicitation. Part of the Polish Sign Language Corpus is publicly available via an open online repository (Kuder et al., 2022; Wójcicka et al., 2020). For the present study, we selected 20 recordings of free conversation. The dataset includes signing by 18 deaf signers. The age distribution of the signers is as follows: 3 signers aged 18–30, 9 signers aged 31–45, 2 signers aged 46–60, and 4 signers aged 60+.

### Annotation

In each dataset, we manually identified instances of negative headshakes by searching the sign gloss annotations and translations to find negative sentences, and subsequently using visual inspection of the video to locate the headshake (if present in the sentence). For each headshake, we annotated on newly created tiers in ELAN (a) whether it was spreading or non-spreading, and (b) whether it spanned any manual negation sign or not. We defined these labels as follows:

**spreading headshake**: A headshake which overlaps with at least two manual signs.
**non-spreading headshake**: A headshake which overlaps with a single manual sign only, whether or not the headshake extends over some period of manual non-activity before or after the manual sign.
**with manual negation**: A headshake which overlaps with at least one manual negative sign.
**without manual negation**: A headshake which does not overlap with any manual negative sign, whether or not a negative sign occurs in the vicinity of the headshake (e.g., in the same sentence).

The definitions of these labels are also illustrated schematically in Figure 5. We discuss the consequences of these definitions in the Results and Discussion sections.

**Figure 5.**
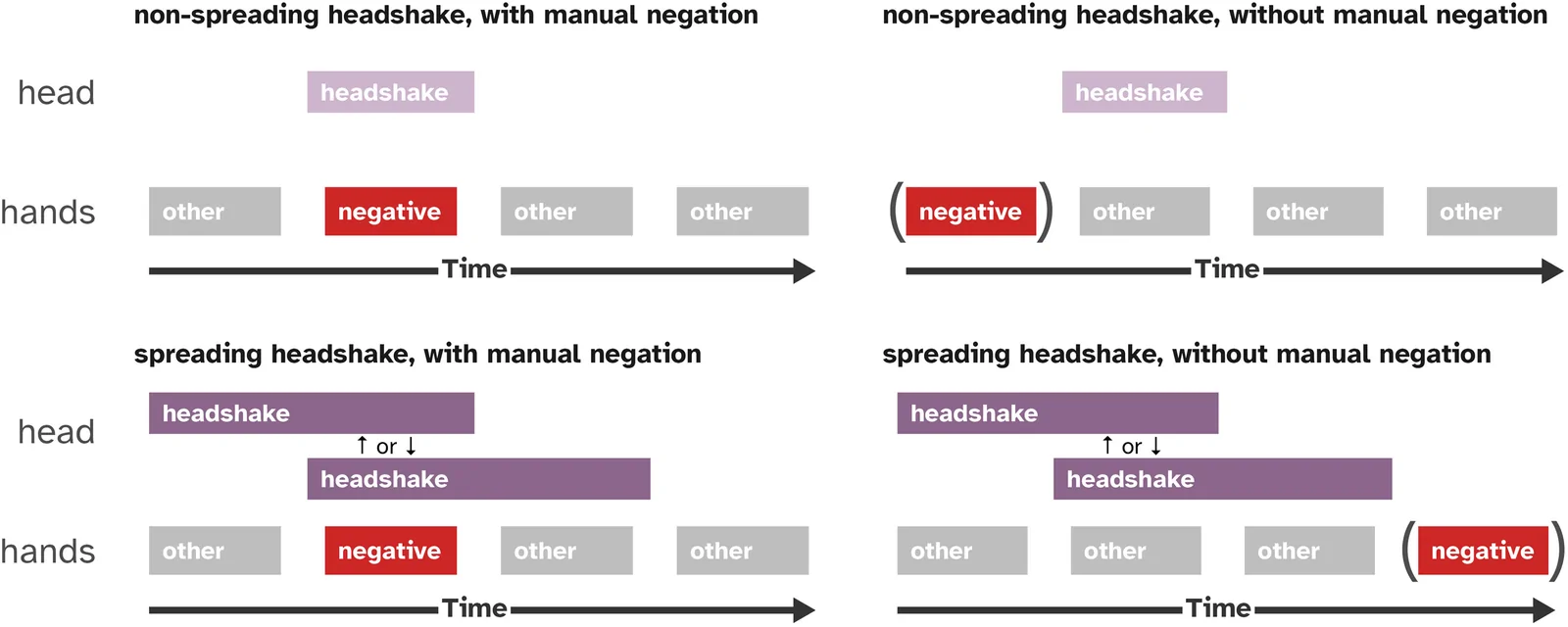
Schematic representation of spreading vs. non-spreading negative headshakes with or without overlapping manual negation.

Finally, for each manual negative sign that co-occurred with a headshake, we assigned a category label, classifying it into *general clause negator* (e.g., not), *negative possessive* (have.not), *negative word* (e.g., nobody, nothing, never), *negative modal* (e.g., cannot, must.not, not.allowed), *negative verb*, which included both verbs expressing a negative meaning in a single sign (e.g., refuse) and verbs with a morphological/affixal expression of negation, and *other* (signs which cannot be classified into one of these types). For this, we relied on existing descriptions of negation in these languages, as well as on the glosses.

The annotation was done separately for each dataset by different annotators, but the first author has revised all the annotations in each dataset.

The labels of spreading vs. non-spreading headshake and manual vs. no manual negation rely on a very simple rule of co-occurrence with other signs (that had already been annotated in the data) as described above (see Figure 5), and annotations were also double-checked by the first author. A sign is treated as co-occurring with headshake if the headshake spreads over at least the stroke phase (the main motion) of the sign; a spreading headshake thus co-occurs with at least the stroke phases of at least two signs.

The category labels for manual negative signs were based on language-specific rules for each lexical negative sign. The annotations for the lexical negative signs were already present in the dataset. Nevertheless, alternative classifications of specific negative signs into classes are possible, and therefore further more focused language-specific analyses are needed.

For the issue of identifying headshakes, some studies have shown that human annotators are reliable in identifying this nonmanual marker (Esselink et al., 2024; Kousidis et al., 2013). In addition, we inspected the headshakes graphically after computer vision processing by plotting the yaw rotation values, expecting to see a periodic oscillation pattern reflecting the turns of the head, which provides an additional layer of controlling that there is, in fact, a headshake happening.

To test whether annotation of the headshake boundaries was reliable across datasets, three of the annotators annotated the same subset of 100 headshakes from the German Sign Language corpus.^9^ One of the annotators was the original annotator of this dataset, and the other two were the annotators for the Polish Sign Language and Brazilian Sign Language datasets. These additional annotators were given the approximate region of occurrence of the headshake, and were tasked with identifying the boundaries of each occurrence. The resulting annotations and analysis script can be found in the linked project repository (see Data Availability Statement).

The mean Jaccard index (i.e., intersection over union) for the pairs of annotators are 0.76, 0.79, and 0.79. The mean differences in duration between the pairs of annotators are 1.3, 1.9, and 3.2 frames at 50 fps; the differences at 25 or 30 fps (which is the case for the other languages in the study) would be proportionally smaller. This means that while the annotators are not perfectly aligned, the differences between the annotators are minor, also relative to the differences in headshake duration we observed between the languages. For example, the estimated difference in length between headshakes in German Sign Language vs. Kata Kolok is 530 ms on average, and the estimated average effect of spreading on duration is 550 ms, both corresponding to over 13 frames at 25 fps. We thus can be quite confident that the differences in duration we find are not due to annotation errors. In the future, it would be desirable to automate headshake boundary annotations, but this problem has not yet been solved.

### Computer Vision Processing and Post-Processing

We use 3DDFA_v2 (Guo et al., 2018, 2020) to extract the rotation angles of the head (pitch, yaw, and roll). 3DDFA_v2 is a model-based method that fits a 3D Morphable Model (3DMM), an average 3D head that can be adjusted in shape and rotated in space, to each video frame, and extracts the head’s pitch, yaw and roll from the orientation of the fitted model. Because it fits a full 3D head rather than tracking a set of visible points, it can estimate head orientation even when parts of the face are turned away from the camera or covered. It predicts the model parameters in a single pass with a lightweight network, and is trained to stay stable across consecutive frames by generating short artificial video clips from still images during training, which makes it well suited to frame-by-frame video analysis.

Previous research on headshake and head nods in sign languages has relied on OpenFace (Baltrusaitis et al., 2018) or MediaPipe (Lugaresi et al., 2019), which are landmark-based methods; our first attempt at analyzing the current data has also used OpenFace. However, despite the fact that these instruments produce reasonable results and can be used in analyzing head movements in sign languages (Sargano et al., 2024), we have discovered an issue that they suffer from, while 3DDFA_v2 performs much better in this respect, namely angle sensitivity (see end of this section for further details).

For the headshake, we used the yaw measurement that 3DDFA_v2 outputs as the measure of head rotation. While it is true that headshake can also involve additional rotation on the other axes, we focused on only one axis for simplicity, and leave exploration of more complex movements for future research. For future analysis of other negative head movements, like backward head tilts, it will be necessary to include other rotation measures.

Because 3DDFA_v2 measurements of rotation are noisy, and we were interested in the frame-by-frame dynamics of head movements, we applied spline-based smoothing to the raw yaw values before calculating the other measures—see Kimmelman et al. (2024) for an initial presentation of the smoothing algorithm and Bulla and Kimmelman (2026) for a detailed discussion.

In short, spline-based smoothing estimates a smooth regression function by minimizing a penalized least-squares criterion that balances fitting to the observed data and a penalty on excessive wiggliness. Following Reinsch (1967) and Wahba (1990), the smoothing spline $\hat{f}$ solves

$$\hat{f}=\arg\;\min_{f}\;\sum_{i=1}^{n}{\left(y_{i}-f\left(x_{i}\right)\right)}^{2}+\lambda \int {\left\{f^{\left(m\right)}\left(t\right)\right\}}^{2}dt,$$

where the roughness penalty ∫{*f*^(*m*)^(*t*)}^2^*dt* controls the curvature of *f*. In our implementation, we fitted quintic smoothing splines with a penalty on the integrated squared third derivative (*m* = 3). This guarantees well-behaved derivatives up to second degree, which is necessary for our peak detection workflow. Furthermore, the smoothing parameter *λ* ≥ 0 governs the bias-variance trade-off: larger *λ* yields smoother fits with higher bias and lower variance, whereas smaller *λ* follows the data more closely at the cost of increased variance (Wahba, 1990). The selection of *λ* is typically carried out by generalized cross-validation (GCV), a computationally efficient surrogate for leave-one-out cross-validation; when splines are embedded in additive-model frameworks, information criteria or REML offer viable alternatives (Wahba, 1990; Wood, 2017). We selected *λ* by GCV via the ss routine in the npreg package (Helwig, 2020, 2024). The resulting differentiable smoothing curve is then numerically differentiated to locate peaks, which can be subsequently classified by a specific set of rules. From a more theoretical perspective, it is noteworthy that smoothing splines are global, unlike local smoothers such as kernel regression. That is, a single smooth function is estimated over the entire domain. The estimator allows for a linear-smoother representation $\hat{y}$ = *S*_*λ*_**y**, enabling efficient computation via banded linear systems and clear model-size control through the effective degrees of freedom edf = *tr*(*S*_*λ*_) (Wahba, 1990).

For the purposes of this study, we only used three measures from this script: (1) robust *amplitude*, a 90th percentile range of the yaw values within a headshake, in degrees; (2) *median velocity*, median absolute change in yaw between consecutive frames within a headshake; and (3) robust *max velocity*, the 90 percentile absolute change in yaw within a headshake. For each instance of a single continuous headshake, we thus calculated one measure each for amplitude, median velocity, and max velocity, respectively, as shown in Figure 6. Note that the velocity measures are calculated in degrees per frame, but then converted to degrees per second for the statistical analysis.^10^

**Figure 6.**
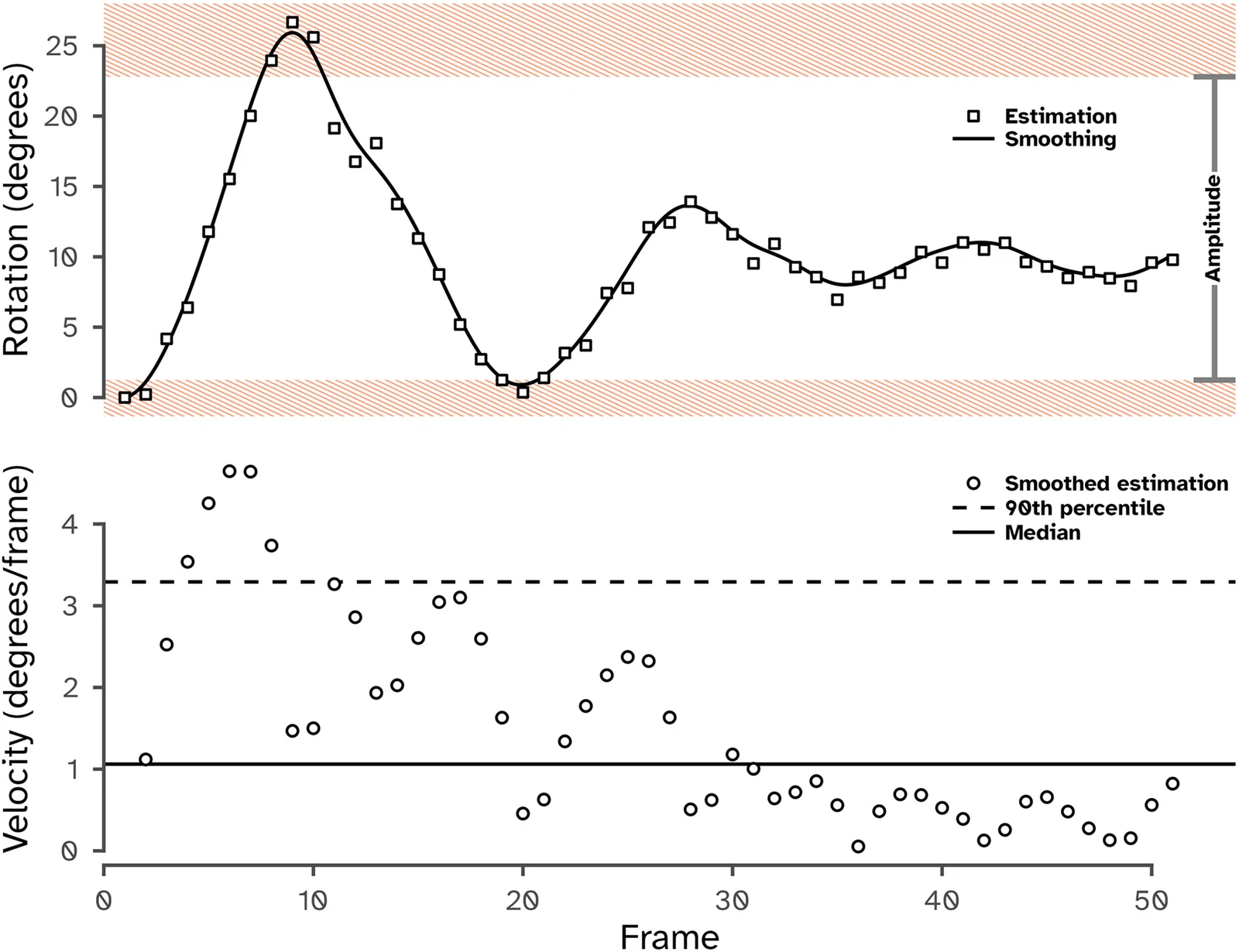
Measurements of headshake yaw rotation from 3DDFA_v2 outputs using a single example headshake from example (1), with multiple rotation cycles spanning 51 frames. The top panel shows the 3DDFA_v2 rotation estimation output per frame as square points and the smoothing of these as a solid line. The top and bottom crossed out areas are outside the 90 percentile range of yaw rotations, thus marking the end points of the yaw rotation amplitude range. The lower panel shows the smoothed velocity of yaw rotations across frames, with the dashed line showing the 90 percentile of smoothed velocities, the operationalized max velocity, and the solid line showing the median velocity.

We chose these measures because, together with duration, they represent the very basic kinematics of headshake, and they complement each other. For instance, a headshake can be large (have a high amplitude) but slow (low median velocity), or large and fast, small and fast, etc. We looked at both median and maximum velocity because a headshake can be very fast in one region, but slow in other regions, so median velocity alone will not capture such dynamics. At the same time, it is clear that these measures are not capturing all the important kinematics of headshake, and also that they are not independent from each other. Therefore, further exploration of the data with other measures, including possible composite measures, is necessary in the future.

To analyze internal headshake kinematics, we divided the headshakes that overlapped with a manual negative sign into three possible zones: pre-negative sign, negative sign, and post-negative sign, based on the annotations for the manual negative sign, excluding the transitional movements. Pre- and post-negative sign zones shorter than two frames were considered to be annotation errors and removed from analysis.

In our dataset, signers in different languages were filmed at different angles. For instance, in the German Sign Language corpus, the signers are filmed at a frontal angle, while in the Russian Sign Language dataset, the signers are filmed at an approximately 45 degree angle, and different signers are filmed from different sides. We decided to test whether computer vision solutions are sensitive to the angle to camera. To do this, we recorded a dataset of 9 people conducting different head rotations (large and small ones), recorded from 4 cameras at 0 to 57 degree angle (see the project repository linked in Data Availability Statement). We then explored head rotation estimation (specifically for yaw, the relevant dimension for our study), comparing the non-frontal cameras to the frontal camera, both in terms of correlation, and in terms of estimated amplitude and velocity measures, and the differences between large and small rotations.

We have discovered that OpenFace estimations of yaw are very strongly affected by the angle to camera, specifically, the estimates of range, amplitude, and velocity all become lower and more noisy with more deviation from the frontal angle. At the same time, 3DDFA_v2 estimates were barely affected by angle, with a slight decrease in estimates only at the 57 degree angle. The differences in overall average estimates between the cameras for the measures are under 5% after smoothing for range and median velocity, and around 10% for robust maximum velocity. However, the estimated differences in amplitude and especially median velocity between large and small movements were different for the different cameras, also for 3DDFA_v2. Thus, in order to further improve comparability of estimates across different cameras, we tested the following solution: we standardized (calculated *z*-scores) of the measures within each camera. This way, we cannot compare general average amplitude or velocity between cameras, but we can still compare the estimated difference between small and large movements between cameras.

We found that, for OpenFace, this does not consistently improve the comparability of estimated measures between the different cameras. However, for 3DDFA_v2, standardization leads to very strong comparability for range and median velocity, with estimates of differences between small and large movements only differing by around 5% between the different cameras. For the robust maximum velocity, non-standardized scores show differences between angles below 5%.

On the basis of this testing, we concluded the following. Despite the differences in angles of recording between the different datasets, we can compare them to each other using head rotation estimation from 3DDFA_v2 directly in terms of robust amplitude, median velocity, and robust maximum velocity. We thus build models using the estimates of these measures without standardization first. However, for additional control of comparability of the effects of other variables, like spreading, and for comparing such effects between languages, we also separately build models using standardized amplitude and velocities. In these models, we by design expect no effect of languages, but potential interactions between language and other variables. If the models disagree, we put more trust in standardized models for the estimation of the main effects other than the effect of language.

### Statistical Analysis

The full details of our statistical analysis, including the data and scripts that can be used to reproduce the results, are linked in Data Availability Statement.

We used R v4.6.0 (R Core Team, 2026) in RStudio (Posit Team, 2025) for all data processing and statistical analysis. We relied on the following packages: tidyverse v2.0.0 (Wickham et al., 2019) for data processing and visualization; here v1.0.2 (Müller, 2020) for path and file management; lme4 v2.0.1 (Bates et al., 2015) and lmerTest v3.2.1 (Kuznetsova et al., 2017) for fitting mixed effect linear regression models; marginaleffects v0.32.0 (Arel-Bundock et al., 2024) for interpreting the models; tidysigns v0.2.2 (Börstell, 2025) for processing ELAN (.eaf) files; ggdist v3.3.3 (Kay, 2024), ggrepel v0.9.6 (Slowikowski, 2016), patchwork v1.3.2 (Pedersen, 2025), rnaturalearth v.1.2.0 (Massicotte & South, 2017), and scales v1.4.0 (Wickham et al., 2025) for visualization.

For statistical modeling, we generally proceed as follows: we fitted a linear mixed effects model with i) the variable of interest (headshake duration/amplitude/median velocity/maximum velocity) as the outcome; ii) linguistic, sociolinguistic, and kinematic factors as fixed predictors, and iii) signer as a random intercept. We started from a null model (without covariates), and apply forward stepwise model selection using AIC for comparing non-nested models and Log Likelihood for nested models to see if adding the dependent variables (and their interactions if considered relevant) improves the model. We also explored the residuals of the selected models to examine whether the model assumptions are met. For all the models, it turned out that non-transformed outcomes lead to non-normally distributed residuals. Therefore, we applied a log transformation to these measures for improving model fit, and proceed through model selection with the transformed outcomes.

## RESULTS

### Overview of Headshake in the Dataset

As discussed above, the dataset contains 2,370 instances of headshake, which are not equally distributed between the languages (see Figure 4). In all the languages, we found both spreading and non-spreading headshakes, and both headshakes that overlap with a manual negative sign and headshakes that do not. Note that, in the “manual” category, we include both headshakes overlapping with the general manual negator like not and with other manual negative signs.

Figure 7 shows the distribution of headshakes in the dataset with and without an overlapping manual negation. Several observations can be made. First, the languages that have been described as manual dominant in some publications, namely Russian Sign Language, Kata Kolok, and Brazilian Sign Language, indeed have the highest proportion of cases where the headshake overlaps with a manual negative sign (79–91%). Second, even in these languages, there are some cases of headshakes that do not overlap with a manual negative sign. Finally, in all languages, headshakes overlap with a manual negative sign more often than not, with the lowest proportion belonging to Dutch Sign Language, in which 55% of the negative headshakes overlap with a manual negation. Even in the nonmanual dominant sign languages, it appears that manual negative signs are very frequent. Note, however, that in our definition, the cases of manual negation include both basic clause negation (which is claimed to be optional in nonmanual dominant sign languages, but mandatory in manual dominant sign languages) and other types of manual negative markers, such as negative adverbials, which should not be optional in any type of language, because they contribute additional meaning.

**Figure 7.**
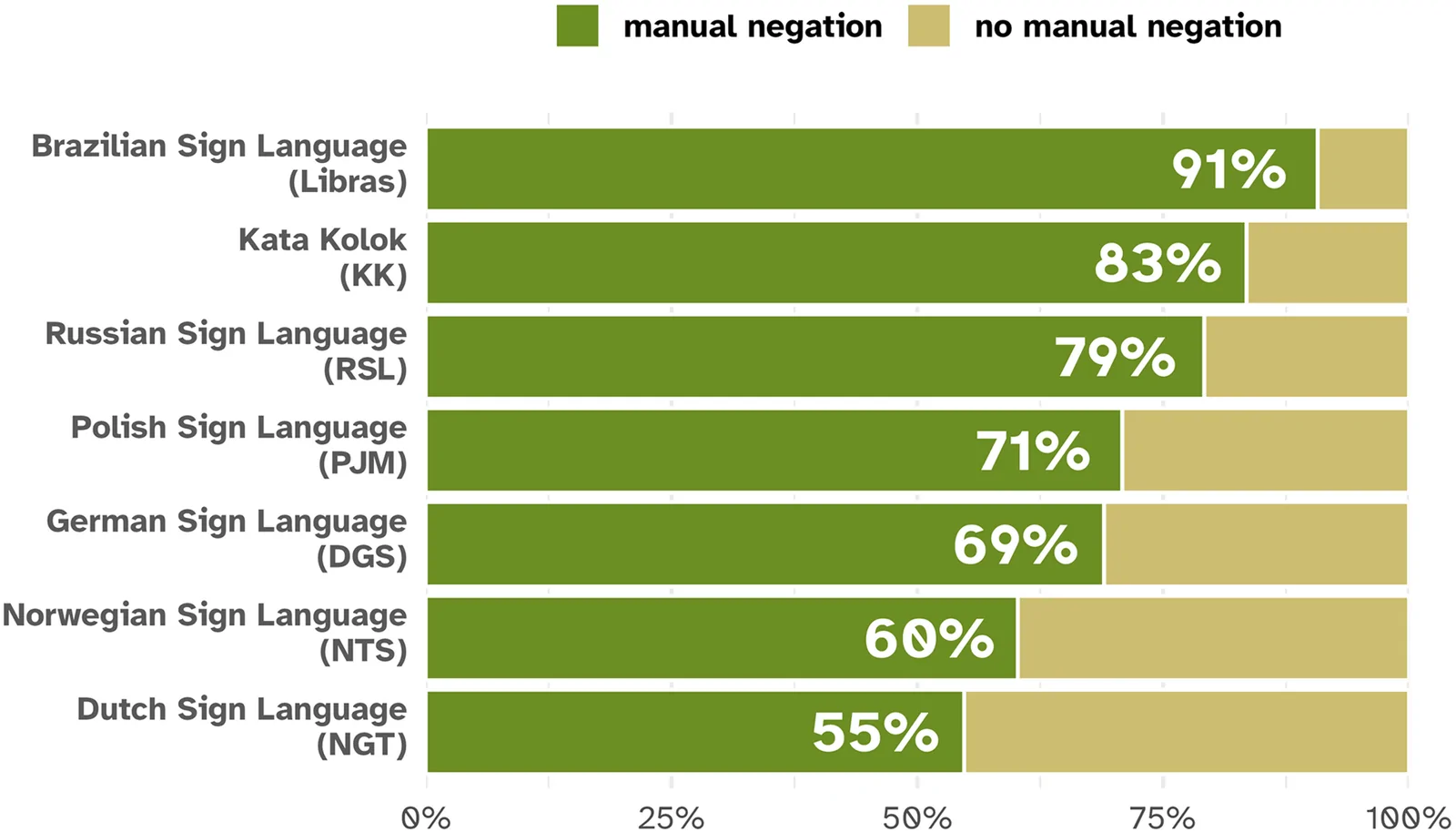
Distribution of headshakes with and without an overlapping manual negative sign of any type by language. Both general negation manual signs, and other manual negative sign types are included in the count.

Returning to the issue of headshakes that do not overlap with manual negative signs in the manual dominant languages, we can observe one pattern that is especially common in Russian Sign Language, but also found in a few cases in German Sign Language and Norwegian Sign Language, namely that the negative sentence does in fact contain a negative manual sign, which, however, is not overlapping with the headshake. Instead, the headshake in such cases either starts after the manual negative sign, or ends before it, as in (13) above. By our definition, such cases are classified as “no manual negation”, and we continued to analyze them as such as we focused on the headshakes themselves, even though a manual negative sign is present in their vicinity. Examples of exclusively nonmanual negation in Russian Sign Language, where there is no manual negative sign in the sentence, are very few, but we still analyze them, too.

Figure 8 shows the distribution of spreading vs. non-spreading headshakes in the dataset. Here, we can observe that both spreading and non-spreading headshakes can be found across all languages, but that the proportions vary. Thinking of the previously established association between manual dominance and non-spreading headshakes, we can see that some languages align with this pattern, while others do not. Dutch Sign Language is the most nonmanual dominant in terms of occurrences of manual negative signs,^11^ and it also has the most headshake spreading (81%). Kata Kolok is one of the hybrid languages, and it has the fewest instances of spreading headshake (33%). At the same time, Russian Sign Language is hybrid with a tendency to almost always use a manual negative sign, but shows a relatively high proportion of spreading headshakes (61%).

**Figure 8.**
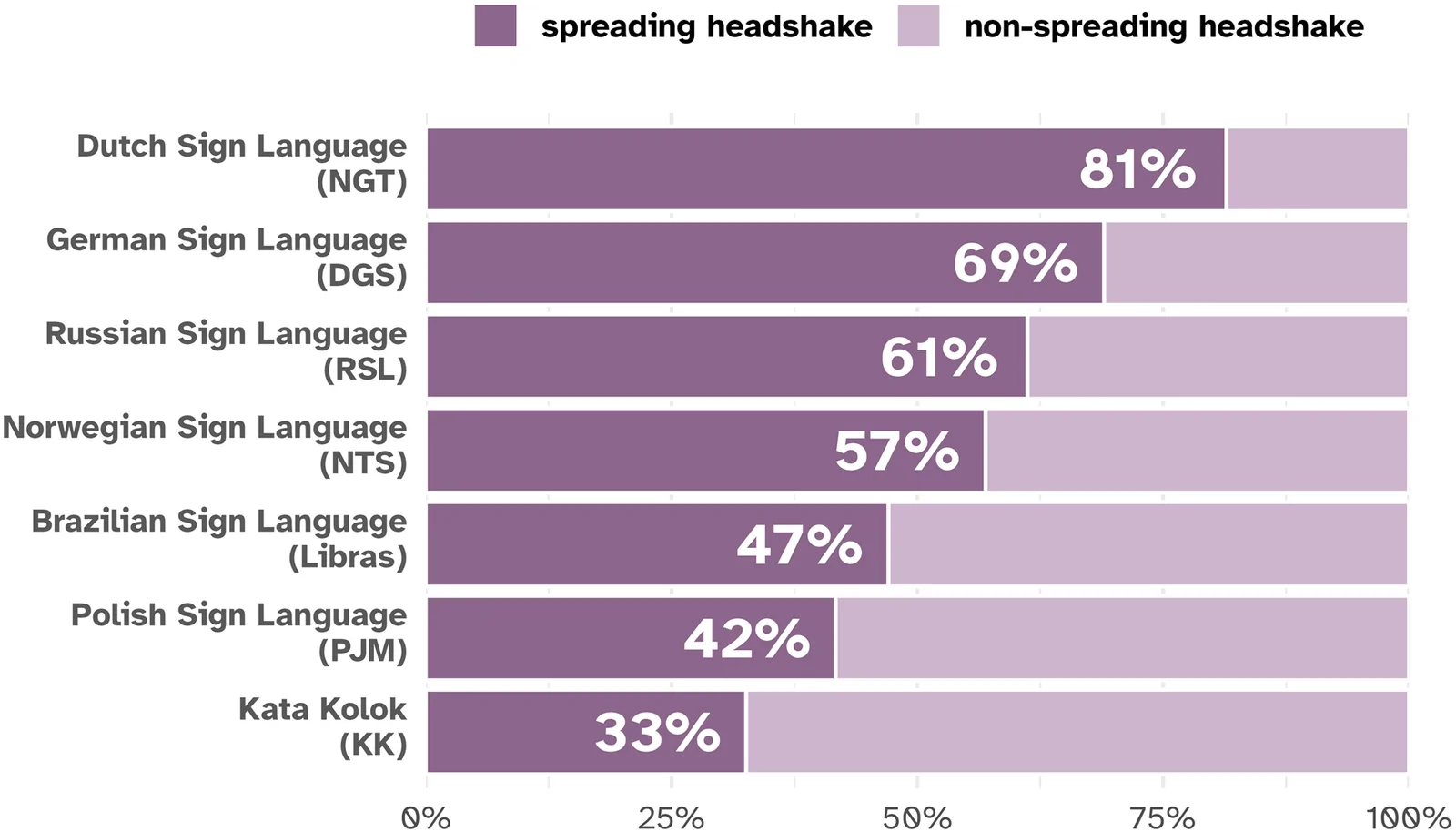
Distribution of the spreading vs. non-spreading headshakes by language.

### General Kinematics of Headshake

We first analyzed how headshake duration is affected by the two linguistic factors (spreading and the presence of an overlapping manual negative sign), and whether it varies across languages. We also added the age of the signer as a predictor, as age has been shown to positively correlate with the duration of manual signs (Börstell et al., 2016, 2024). Visual inspection of Figure 9 indicates that spreading headshakes are longer (as expected), and that Kata Kolok has shorter headshakes than the other languages. The presence of an overlapping manual negative sign does not seem to have a clear effect on headshake duration.

**Figure 9.**
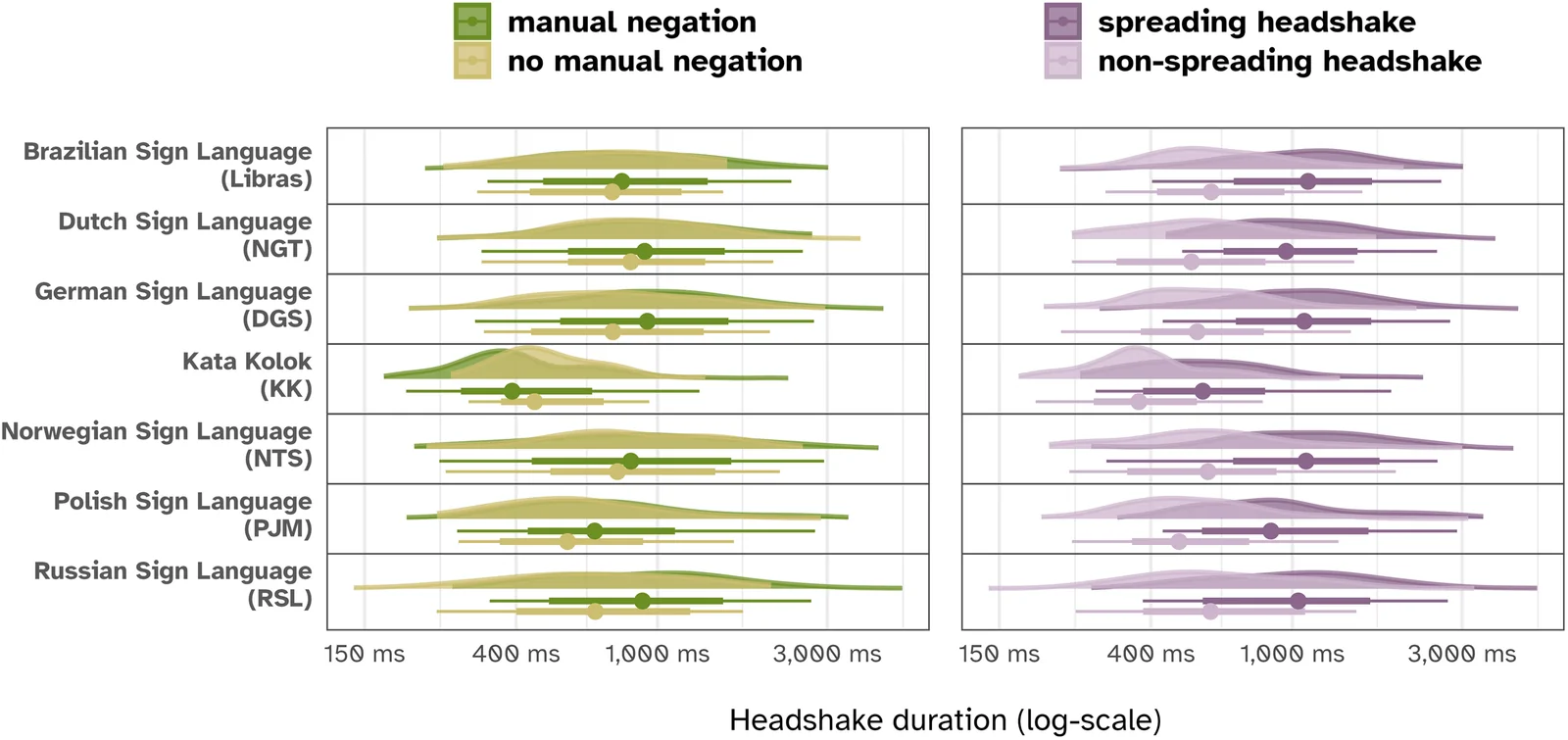
Headshake duration as affected by co-occurrence of manual negation and whether the headshake is spreading.

We fitted a model with log-transformed duration as the outcome. The model shows that language and spreading were significant predictors. The presence of a manual negative sign did not significantly affect the outcome. The full details of the analysis can be found in the project repository.

Spreading headshakes are significantly longer than non-spreading headshakes (estimate = 0.569, *p* < 0.001, which means that spreading headshakes are predicted to be 77% longer). For the language variable, post-hoc pairwise comparison shows that Kata Kolok has a significantly shorter headshake duration than all the other languages; in addition, Polish Sign Language has a significantly shorter headshake duration than German Sign Language, Brazilian Sign Language, Dutch Sign Language, and Norwegian Sign Language.

Adding the interaction between language and spreading does not lead to a significant improvement to the model, so we do not have evidence that spreading affects duration differently across different languages.^12^

For amplitude, median velocity, and max velocity, we fit models both with and without standardizing the outcome within each language, as discussed above. We first discuss the best model without standardization, and then the best model with standardization. The variables we explore are spreading, presence of a manual negative sign, language, age, and interaction between language and spreading. Since amplitude and velocity can be affected by headshake duration, we included duration as a possible predictor as well.

Figure 10 shows that, in all languages except for Russian Sign Language, spreading headshakes have a slightly higher amplitude than non-spreading headshakes, and that average amplitude varies across languages.

**Figure 10.**
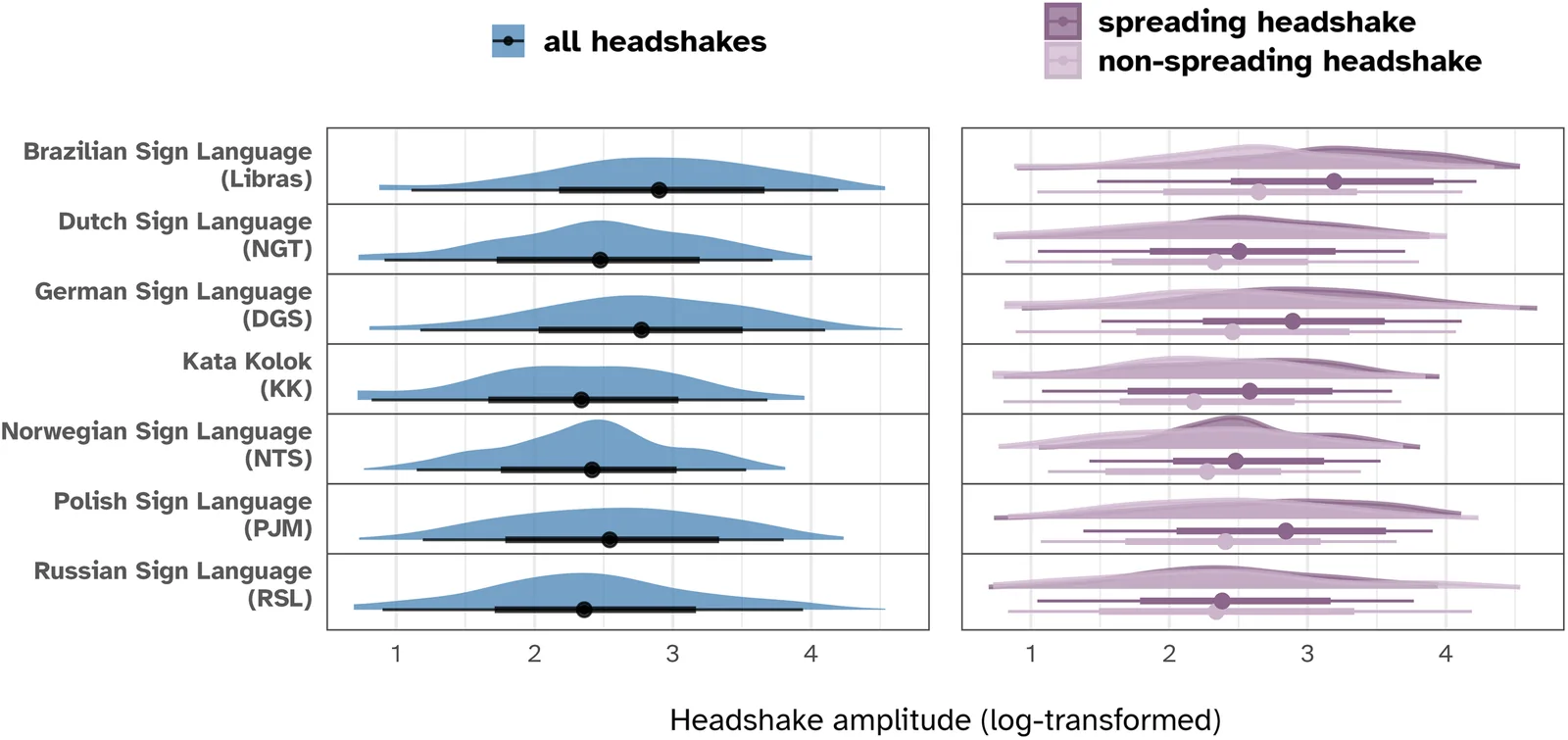
Amplitude as affected by language and whether the headshake is spreading.

The best model for robust amplitude without standardization includes the following predictors: duration, language, spreading, and the interaction between language and spreading. As expected, higher duration of headshakes corresponds to higher amplitude (estimate = 0.0004 per ms, *p* < 0.001). Furthermore, spreading has a significant positive effect on amplitude (estimate = 0.06, *p* = 0.005). Interestingly, languages differ from each other in the estimated mean amplitude, see model predictions in Figure 11. Specifically, Brazilian Sign Language and German Sign Language have the highest amplitudes, while the other languages have lower amplitude. Several of the pairwise differences are significant (after correction for multiple comparisons), such as the differences between Brazilian Sign Language and Kata Kolok, Norwegian Sign Language, Polish Sign Language, and Dutch Sign Language, and between German Sign Language and Kata Kolok and Norwegian Sign Language.

**Figure 11.**
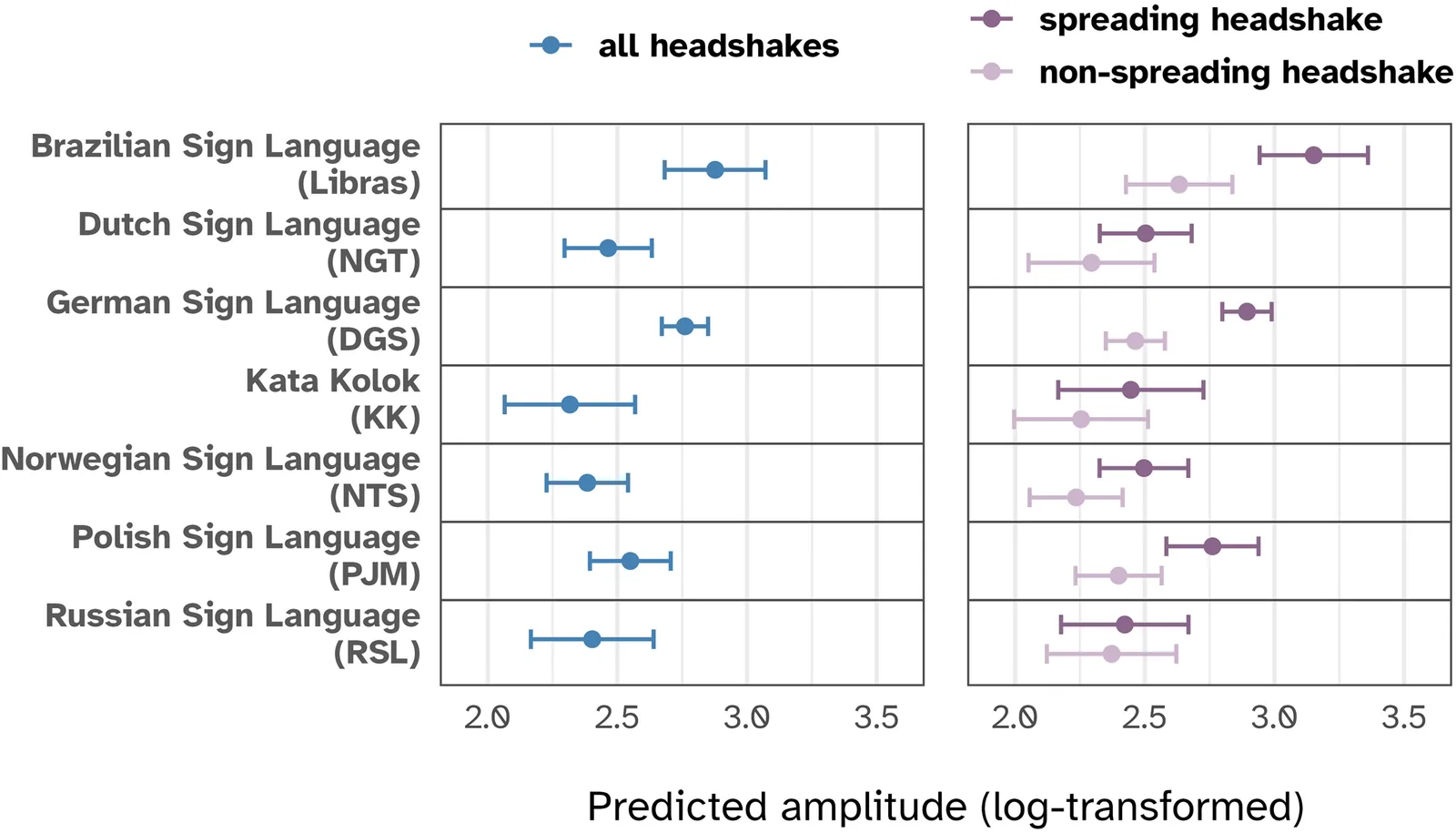
Model predictions for the effect of language and spreading on amplitude with estimates (points) and 95% confidence intervals (whiskers).

In addition, the model includes a significant interaction between the effects of language and spreading, see model predictions in Figure 11. Exploring the model predictions, we can see that the positive effect of spreading on amplitude is largest in German Sign Language and Brazilian Sign Language, and smallest in Russian Sign Language, where there is basically no difference in amplitude between spreading and non-spreading headshakes. The difference in the effect of spreading between Russian Sign Language and Brazilian Sign Language is significant (after correction for multiple comparisons, estimate = 0.36, *p* = 0.01).

In the model with standardized amplitude, we obtain very similar results. Duration, spreading, and the interaction between spreading and language are significant predictors. Importantly, we observe the same pattern of interaction between language and spreading, with Brazilian Sign Language and German Sign Language showing the biggest effect of spreading, and Russian Sign Language showing basically no effect of spreading. The difference between Brazilian Sign Language and Russian Sign Language is significant in this model, too.

For median velocity, the best model without standardization only includes language as a significant predictor. Specifically, it appears that Dutch Sign Language and Norwegian Sign Language have a lower average median velocity than the other languages. Pairwise comparison indicates the following differences as significant: German Sign Language vs. Dutch Sign Language, German Sign Language vs. Norwegian Sign Language, Brazilian Sign Language vs. Dutch Sign Language, and Brazilian Sign Language vs. Norwegian Sign Language. Spreading is not a significant predictor of median velocity.

In contrast, the best model for median velocity with standardization includes only a small positive effect of spreading as significant (estimate = 0.08 log deg/ms, *p* = 0.05). Because the models with and without standardization disagree, it is difficult to interpret the results. However, since in our testing, the absolute estimates of velocity are not strongly affected by angle to camera, while the effects within language are better measured with standardization, we provisionally accept that the data indicates both absolute differences in velocity between the languages, and a small positive effect of spreading on velocity.

For robust maximum velocity, the best model without standardization includes spreading and language as significant predictors. Spreading headshakes have a higher maximum velocity (estimate = 0.06 log deg per ms, *p* = 0.02). In addition, as with median velocity, Norwegian Sign Language and Dutch Sign Language have the lowest estimates, and the differences between them and German Sign Language and Brazilian Sign Language are significant.

The best model with standardization includes spreading as a significant predictor: spreading headshakes have a higher maximum velocity. The two models thus agree in that spreading headshakes have higher maximum velocity.

To sum up, for robust amplitude, median velocity and robust max velocity, we found evidence that spreading headshakes have larger values of these measures than non-spreading headshakes. Amplitude and maximum velocity are also positively correlated with headshake duration, but the effect of spreading is still present when including the effect of duration. The models with non-standardized measurements also show clear cross-linguistic differences, with German Sign Language and Brazilian Sign Language having especially larger and faster headshakes, and Dutch Sign Language and Norwegian Sign Language having slower headshakes. Finally, we also find a significant interaction between the effects of spreading and language on amplitude, namely we observe that the effect of spreading is largest in German Sign Language and Brazilian Sign Language, and smallest (probably non-existent) in Russian Sign Language.

For all the comparisons above, we found that the factor of the presence of a manual negative sign does not significantly affect the outcome. However, for three of the sign languages in the dataset (Kata Kolok, Russian Sign Language, and Brazilian Sign Language), the cases of headshakes without an overlapping manual negative sign are exceptional, and thus, we proposed that these languages should not be included when this factor is considered.

We then separately explored the four sign languages that clearly do use nonmanual-only negation relatively frequently (Dutch Sign Language, German Sign Language, Norwegian Sign Language, and Polish Sign Language^13^), to see whether the presence of a manual negative sign is a significant predictor of headshake amplitude and velocity in this group. Statistical analysis shows that, for this group, the presence of the manual negative sign is not a significant predictor for either kinematic measure, contrary to our hypothesis.

We also hypothesized that headshake kinematics can be affected by the type of negation expressed in the sentence. To operationalize negation type, we annotated the category of the manual negative sign, as discussed above, and focused on the following major types: general negator (e.g., not or neg), negative modal (e.g., cannot), negative possessive (e.g., have.not), negative verb (e.g., give.not or give-neg), and negative word which includes negative pronouns (e.g., nobody) and negative adverbials (e.g., never). We excluded cases that cannot be classified into one of these categories from further statistical analysis. Since Kata Kolok only uses a single general negator (see Lutzenberger et al., 2022), we excluded it from this analysis. Furthermore, we only focus on the headshakes co-occurring with a manual negative signs. This leaves 1,284 headshakes distributed over six languages.

Statistical analysis shows that headshake duration is significantly affected by the manual negative sign category. Visual inspection of the model predictions indicates that headshakes accompanying negative words have the longest duration, and general negators and negative modals the shortest. The difference between negative words and general negator is significant. The fact that negative sign type can affect headshake duration is not surprising, since different negative signs themselves have different durations.

More importantly, statistical analysis shows that the manual negative sign type is not a significant predictor of amplitude, median velocity, or maximum velocity in our data. The full details of the analysis can be found in the project repository.

#### Summary.

We found that, unsurprisingly, headshake duration is affected by spreading and varies across languages. We also found that headshake amplitude and median and maximum velocity are positively affected by spreading (even when controlling for the effect of duration). We also found significant and clear differences in amplitude and velocity between the languages, and, for amplitude, a significant interaction between language and spreading. We did not find evidence of the presence of a manual negative sign affecting headshake kinematics, neither for the whole dataset, nor separately for the languages in which nonmanual-only cases frequently occurred. Finally, while the type of negative sign may affect headshake duration, we found no evidence that it affects headshake amplitude or velocity.

### Internal Kinematics of Headshake

In order to analyze the internal dynamics of headshakes, we divided headshakes containing manual negative signs and spreading into two or three zones: the *negative sign zone* (corresponding to the scope of the negative sign annotation), the *pre-negative sign zone* (the part of the headshake up to the onset of the manual negative sign), and the *post-negative sign zone* (the part of headshake following the offset of the manual negative sign). This allows us to test the two alternative hypotheses we outlined previously (see Figure 3): Is headshake kinematically stronger on the manual negative sign relative to elsewhere, or does it follow a consistent prosodic contour in our languages?

The relevant measures are robust amplitude and median and robust maximum velocity. As with the general headshake kinematics, we model both non-scaled and scaled measures; however, in this case, the scaled models always identify the same significant factors as the non-scaled models, with the exception of the main effect of language, so we do not further discuss these results. The full analysis can be found in the project repository.

Note that in the models discussed in this section, the confidence intervals for the estimates are quite wide for most languages (with the exception of German Sign Language, which has the most data points), so the results discussed here should be treated with some caution.

For amplitude, statistical analysis shows a significant effect of zone duration, significant differences between the zones, significant differences between languages, and a significant interaction between zone and language. The model predictions are visualized in Figure 12. The post-hoc pairwise comparison of the main effect of zones shows that the pre-negative sign zone has a significantly higher amplitude than the other two zones, but also that the post-negative sign zone has a significantly higher amplitude than the negative sign zone. We can observe differences between languages in relation to zones. In most languages, the pre-negative sign zone has a clearly higher amplitude, but in Norwegian Sign Language, the post-negative sign zone has the highest amplitude, and in Brazilian Sign Language, the pre- and post-zones are very close. In addition, in most languages, the negative sign zone clearly has the lowest amplitude, but in German Sign Language, the negative sign zone and the post-negative sign zone are very close in amplitude.

**Figure 12.**
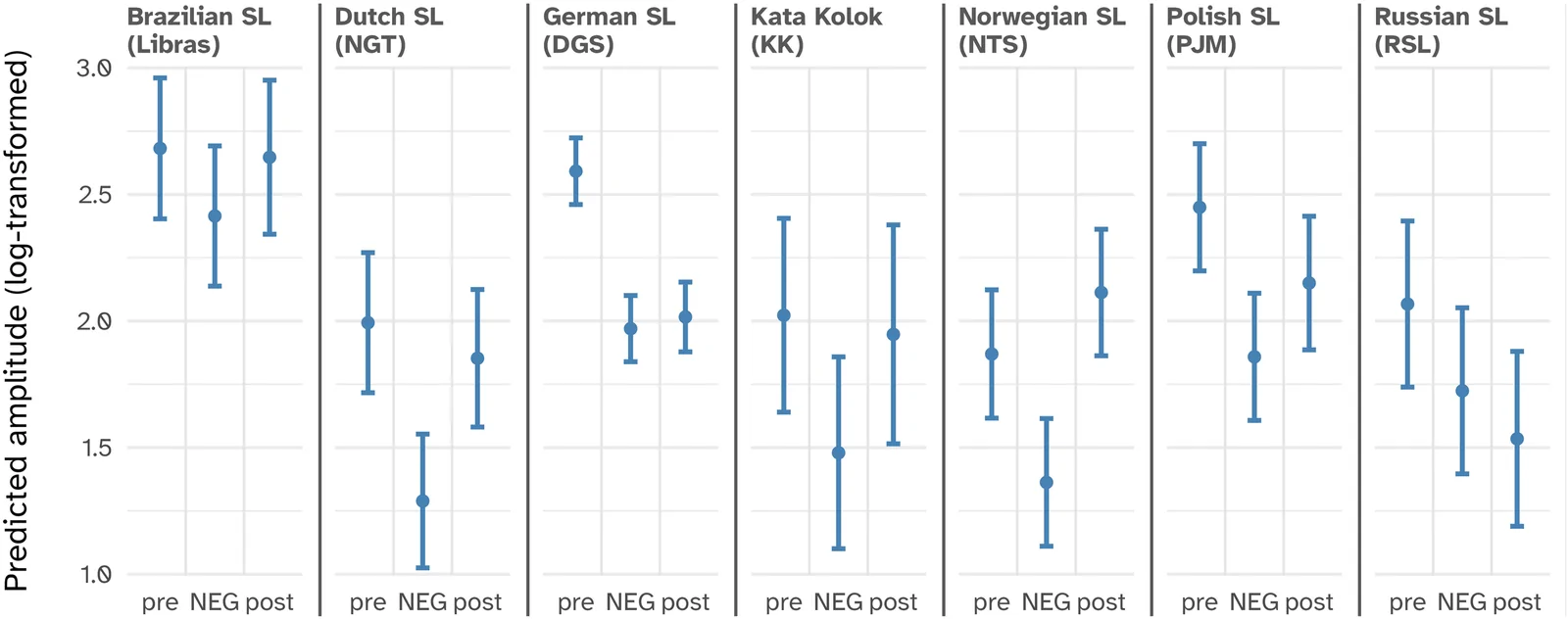
Model predictions for the effects of zone on amplitude with estimates (points) and 95% confidence intervals (whiskers).

To explore the language-zone interaction, we conducted post-hoc tests specifically for the differences in zone effects between Norwegian Sign Language and German Sign Language, the languages most clearly demonstrating different patterns. We found that the difference between the negative sign and post-negative sign zones is significantly different for the comparison between Norwegian Sign Language and German Sign Language (estimated difference = 0.38, *p* = 0.01).

For median velocity, statistical analysis shows that it is positively correlated with the duration of the zone, and there are significant differences between languages (as expected based on the general kinematics analysis). In addition, zones are significantly different: post-hoc pairwise comparison shows that each difference is significant, and specifically that the pre-negative sign zone has the highest median velocity, and the post-negative sign zone has the lowest median velocity. Finally, the analysis also shows that there is a significant interaction between language and zone, so languages behave differently from each other with respect to zone effects. Visual exploration of the predictions of the model (Figure 13) shows that in some languages, like German Sign Language and Russian Sign Language, we observe the order of *pre-negative sign* > *negative sign* > *post-negative sign*, while in other languages, like Brazilian Sign Language and Polish Sign Language, the median velocity of the headshake on the negative sign and post-negative sign zones is very close, or the post-negative zone has even higher median velocity than the negative sign zone.

**Figure 13.**
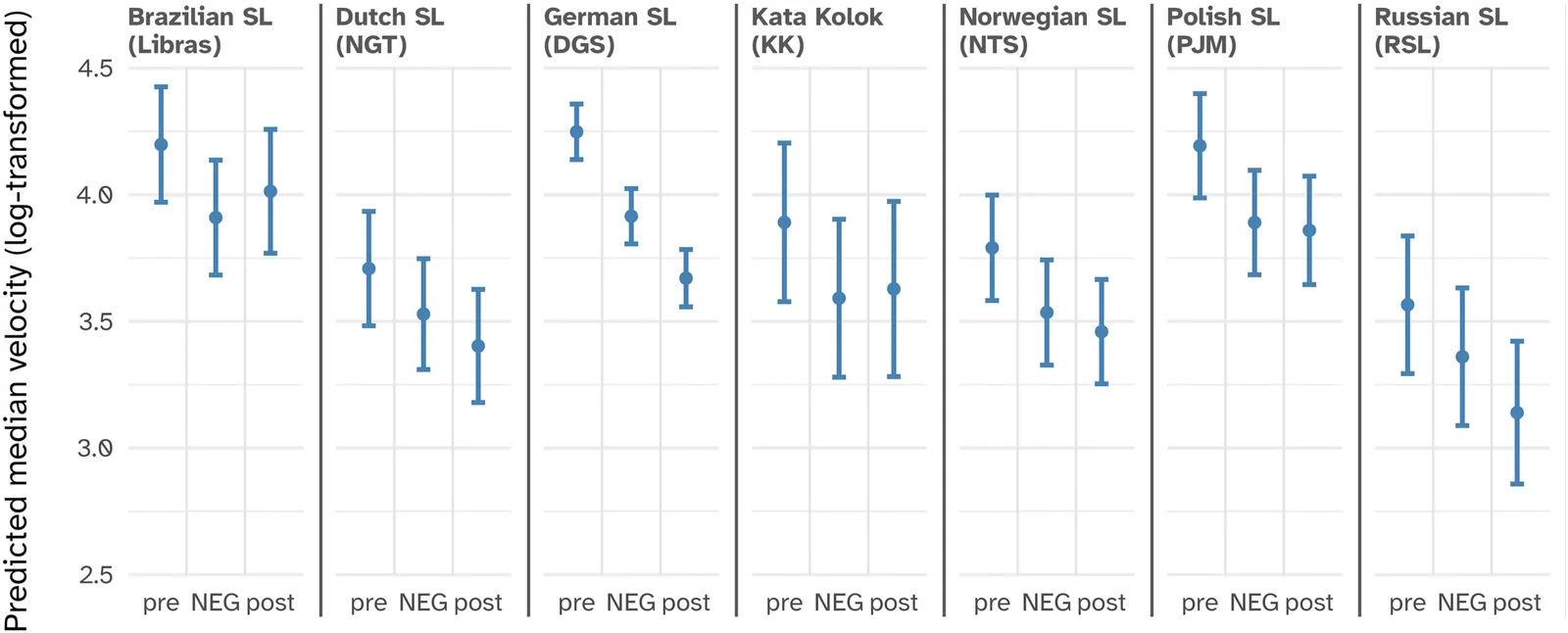
Model predictions for the effects of zone on median velocity with estimates (points) and 95% confidence intervals (whiskers).

To explore this language-zone interaction, we conducted post-hoc tests specifically for the differences in zone effects between Brazilian Sign Language and German Sign Language, the two languages most clearly demonstrating the two patterns. We found that the difference between the negative sign zone and the post-negative sign zone is significantly different in the two languages (estimated difference = 0.26, *p* < 0.01).

For maximum velocity, statistical analysis also shows a significant effect of zone duration, significant differences between languages, significant differences between the zones, and a significant interaction between zone and language, as can be observed in Figure 14 with model predictions.

**Figure 14.**
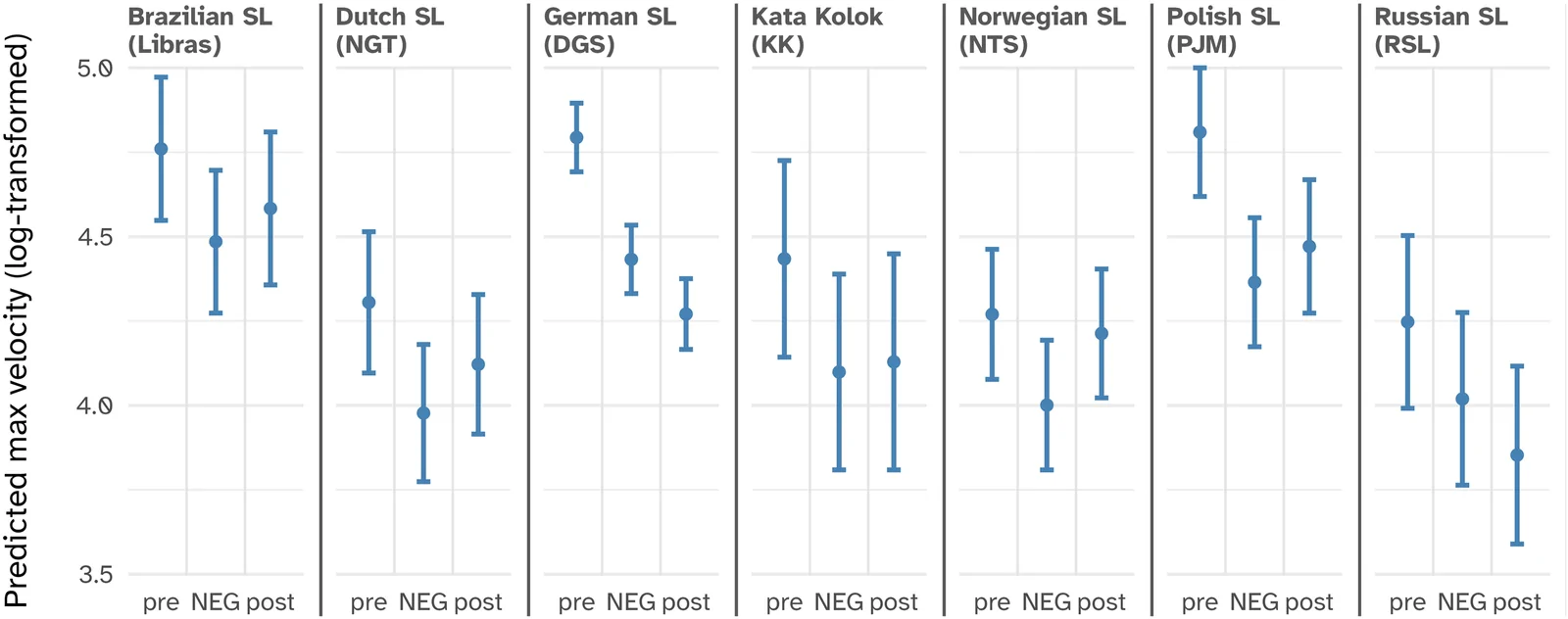
Model predictions for the effects of zone on maximum velocity with estimates (points) and 95% confidence intervals (whiskers).

The post-hoc pairwise comparison of the main effect of zones shows that the pre-negative sign zone has a significantly higher maximum velocity than the other two zones, but also that the post-negative sign zone has a significantly higher maximum velocity than the negative sign zone. Additionally, we again observe here that the languages vary in several ways. In most languages, the pre-negative sign zone has a clearly higher maximum velocity, but in Norwegian Sign Language, the post negative sign zone has a slightly higher maximum velocity. Secondly, in most languages, the negative sign zone clearly has the lowest maximum velocity, but in German Sign Language and Russian Sign Language, the negative sign zone and the post zone are very close in maximum velocity, and, in the latter, the negative sign zone is even slightly higher than the post zone.

To explore this language-zone interaction, we conducted post-hoc tests specifically for the differences in zone effects between Norwegian Sign Language and German Sign Language, the two languages most clearly demonstrating the two patterns. We found that there is a significant difference between the negative sign and the post-negative sign zones for the comparison between these two languages (estimated difference = 0.38, *p* = 0.01).

#### Summary.

We find that when the headshake overlaps with a manual negative sign and spreads across at least one more sign, there are kinematic differences between the zones within the headshake, which vary between languages. For median velocity, we observed the order: *pre-negative sign zone* > *negative sign zone* > *post-negative sign zone*, while for amplitude and maximum velocity, we observed the order *pre-negative sign zone* > *post-negative sign zone* > *negative sign zone*. For both amplitude and the median and maximum velocity, we observed that some sign languages exhibit differences in the kinematic characteristics of these zones. It is worth noting that we observed no language in which the negative sign zone has higher amplitude or velocity than the other zones, and that the pre-negative sign zone usually dominates according to these kinematic measures.

## DISCUSSION

### Headshake Duration

We found that headshake duration varies between the languages in our study. Specifically, in Kata Kolok, average headshake duration is shorter than in all the other languages, and in Polish Sign Language, it is significantly shorter than in German Sign Language, Brazilian Sign Language, Dutch Sign Language, and Norwegian Sign Language. While both Polish Sign Language and Kata Kolok use manual negation frequently, they allow for headshakes to spread, but, as it emerges from our study, the headshake durations are still shorter on average. The effect is especially striking for Kata Kolok, where spreading headshakes have durations comparable to non-spreading headshakes in other languages, and the non-spreading headshakes are significantly shorter still. The finding that headshakes are short in these two sign languages is in line with previous reports (Kuder, 2021; Marsaja, 2008).

We do not have a clear explanation for why Polish Sign Language and especially Kata Kolok have shorter headshake duration than the other languages. For Kata Kolok, we could speculate that its status as a rural sign language might make it typologically different also with respect to the use of negative headshake, but see de Vos and Pfau (2015) and Lutzenberger et al. (2022) for a more nuanced view on the typological features of these languages. In general, we have found clear indications that headshake duration can vary across sign languages, and further typological research is required to explain the factors underlying this variation.

### General Headshake Amplitude and Velocity

We find significant and clear differences between the languages in terms of headshake amplitude and median and maximum velocities. German Sign Language and Brazilian Sign Language have higher values for robust amplitude, while Dutch Sign Language and Norwegian Sign Language have lower values for median and maximum velocities. Several observations can be made.

First, it is interesting to observe that amplitude and velocity are not fully correlated. In terms of amplitude, German Sign Language and Brazilian Sign Language are exceptional, while in terms of velocity, they pattern together with several other sign languages. Thus, it is useful to explore these kinematic measures separately also in future studies.

Second, it is not obvious how these kinematic measure differences can be connected to the typological parameters of manual vs. nonmanual dominance. While German Sign Language is clearly nonmanual dominant, leading us to expect that headshakes would be kinematically strong, both Dutch Sign Language and Norwegian Sign Language are also clearly nonmanual dominant, presenting even higher proportions of cases of nonmanual only negation in our dataset, and still, the headshakes in these languages are weakest kinematically on average. Brazilian Sign Language offers evidence against our hypothesis from the opposite side: it is clearly a hybrid language, with very high proportion of manual negative signs, but with kinematically strong headshakes.

Finally, from a methodological perspective, it is important to note that the differences in strength of the kinematic measures cannot be attributed to differences in angle to camera across the datasets. German Sign Language headshakes are among the strongest kinematically, and the dataset is recorded mostly frontally; however, the Brazilian Sign Language videos are recorded at around 25 degrees, similar to the Dutch Sign Language videos, while kinematically these two languages are very different from each other.

### The Role of Spreading

We hypothesized that spreading headshakes would be longer than non-spreading ones, which should almost by definition be true, and which turned out to be the case. In addition, we hypothesized that, because spreading headshakes are longer, they are visually prominent due to length, and thus might require less kinematic prominence via amplitude and velocity; see Börstell (2024) for a comparable effect in the kinematics of manual signs. However, this turned out not to be the case. In fact, spreading headshakes have higher amplitude, median velocity, and maximum velocity, even when taking duration into account.

We cannot fully explain this pattern using the data from this study. However, we can hypothesize that the explanation lies in the reason why headshakes spread in the first place. It is possible that spreading itself is correlated with more emphatic negation. The causal chain would then be as follows: the signer needs to express negation more emphatically, which causes the headshake to become both longer (i.e., spread over multiple signs) and kinematically stronger, a reasoning that is reminiscent of Jespersen’s cycle (Jespersen, 1917; van der Auwera, 2009). Again, more detailed further research is required to pinpoint the factors causing headshake to spread and how these factors correlate with kinematic properties of headshake.

We can also see parallels between headshake kinematics and prosodic realization of prominence, in both speech and co-speech gestures. For instance, Ambrazaitis and House (2023) find evidence for the multiple redundant expression of prominence in speech and co-speech gesture instead of compensatory mechanisms. They thus argue for the Cumulative Cue Hypothesis, and against the Compensatory Cue Hypothesis. Note that their hypotheses concern prosodic prominence in general, and do not explicitly address the expression of negation.

It is also possible to hypothesize that headshakes need to have a certain minimal amplitude to be perceivable by the addressee, and this holds for both spreading and non-spreading headshakes. According to this logic, non-spreading headshakes do not have to be larger to compensate for the shortness; they only need to be large enough to be perceivable. Further perception studies are necessary to test this hypothesis.

One of the potentially most interesting findings is that there are cross-linguistic differences in the effect of spreading on the amplitude (although we do not find evidence for such differences for the velocities). Specifically, Russian Sign Language is the only sign language which shows no effect of spreading on amplitude, while in all the other languages, spreading headshakes are larger than non-spreading headshakes. Russian Sign Language is one of the languages that is closer to the manual dominant end of the scale, but it is not the only one, and not the most extreme. Thus, we can observe an indication that there might be a typological difference between languages that use stronger headshakes with spreading and those that do not, but that this distinction is not fully correlated with manual dominance. Note, however, that the Russian Sign Language data set has only seven signers, so generalizability of the findings needs further testing.

### The Role of the Manual Negative Sign

We found no evidence that the presence of a manual negative sign affects kinematics of negative headshake in our dataset. This is in line with our expectations for nonmanual dominant sign languages. As has been argued for at least some nonmanual dominant sign languages, headshake itself is the primary marker of negation in languages of this type, and possibly a realization of a morphological negative feature (Pfau, 2016). The manual negative sign does not express negation; instead, it is an element that occurs in negative contexts, but only when the main nonmanual marker is present. In some theories, this has been interpreted as the negative headshake carrying the negative meaning, with the manual sign not being negative by itself, but rather requiring a “true” negative element (i.e., the headshake) to co-occur. Under such an approach, it is not surprising that the kinematics of the headshake is not affected by the presence of a manual negative sign, as the role of the latter is not to convey negation. This theory would also predict that negative headshake in such languages should be (nearly) obligatory, while the use of manual negative signs is optional. Since we did not analyze negation in general in our dataset, but focused on headshake only, we cannot determine whether headshake is obligatory in these languages.

For manual dominant sign languages (or, more precisely in our case, hybrid sign languages), we also did not find evidence that the presence or absence of a manual negative sign affects headshake kinematics, which is contrary to our hypotheses. However, for these languages, we can offer an alternative explanation, namely that the cases of purely nonmanual negation are exceptional and rare, and therefore, we do not have enough data to study them carefully. In some cases, like example (13) above, the manual negative sign does occur in the same sentence with the headshake, so the functional expectation does not hold.

Another surprising outcome is that we did not find evidence that the type of manual negative sign affects headshake kinematics. It is reasonable to expect different types of negation—e.g., sentential vs. constituent—to be accompanied by different types of headshake. However, our labeling for the type of manual negative sign was crude, and based on classifying lexical signs into a small number of predetermined categories for ease of cross-linguistic comparison. We did not analyze how these categories relate to actual differences in the syntactic and semantic structure of negative sentences in these languages. Nor did we look at how negation is expressed in different clause types (e.g., prohibitives, existentials, etc.) that are known to vary in negative structures (Dryer, 1988; Miestamo, 2007). Further analysis of this topic is therefore required.

### Headshake Zones

We had two different sets of expectations for the kinematics of headshake zones within spreading headshakes co-occurring with a manual negative sign (recall Figure 3). On the one hand, the lexical association between the headshake and the manual negative sign predicted the headshake to be kinematically strongest during the realization of this sign, as Kimmelman et al. (2024) found for Dutch Sign Language. On the other hand, based on a prosodic view of headshake, we expected a general downward trend in the kinematic measures from the beginning to the end of a headshake.

Our findings generally favor the latter theory, but not completely. First, we found the following ordering from kinematically strongest to weakest of the zones with respect to the kinematic measures:

▪ Amplitude: pre-negative sign > post-negative sign > negative sign
▪ Median velocity: pre-negative sign > negative sign > post-negative sign
▪ Maximum velocity: pre-negative sign > post-negative sign > negative sign

Thus, the pattern is as expected based on the prosodic view for median velocity, but not for maximum velocity or amplitude, as we did not expect the post-negative sign zone to be kinematically stronger than the negative sign zone.

Secondly, we found cross-linguistic variation for the kinematic measures of headshake zones, and thus divergences from the main patterns. For median velocity, in Brazilian Sign Language and Polish Sign Language, the negative sign and the post-negative sign zones are very close. For maximum velocity, in Norwegian Sign Language, the post-negative sign zone has highest velocity, and in Russian Sign Language and German Sign Language the negative sign and the post-negative sign zones are very close. For amplitude, in Norwegian Sign Language, the post-negative sign zone has the highest amplitude, while in German Sign Language, the negative sign and the post-negative sign zones are very close.

How do we interpret these findings? First, it is important to note that we found cross-linguistic variation in the internal kinematics of headshake. This is an indication that headshake kinematics is grammaticalized, and that grammaticalization happens in different ways across different sign languages.

Second, it seems that neither theory predicts the observed general pattern of the lowest velocity of the negative sign zone. From the prosodic point of view, we would expect the lowest velocity to be in the post-negative sign zone (as we do observe for median velocity), and from the grammatical point of view, we would expect the negative sign zone to be kinematically strong in general. Further research, possibly with other kinematic measures such as frequency, is required to explain this pattern.

Note that there is a discrepancy between our findings for headshake kinematics in Dutch Sign Language specifically, in comparison to Kimmelman et al. (2024), even though the same dataset was used in both studies. Specifically, even though the headshake zones in the current study are not fully comparable to the headshake function labels in Kimmelman et al. (2024), the negative sign showed relatively strong kinematics in the 2024 study, and relatively weak kinematics in the current study (although it was not the kinematically strongest region in either study). This discrepancy is due to methodological improvement in the current study: we now apply a better computer vision solution, smoothing, robust kinematic measures, and log-transforming the outcomes to arrive at a statistically more appropriate model.

It is important to acknowledge two potential limitations of the headshake zone analysis which make the findings reported here preliminary. First, as mentioned in the Results section, the confidence intervals for the effect of zone in the best models reported are quite wide for all the languages except for German Sign Language; therefore, the specific patterns and differences between the languages should be interpreted with caution.

Furthermore, we have found that the duration of the negative sign zone is on average shorter than of the other two zones. On average, the negative sign zone is 326 ms, the pre-negative sign zone is 496 ms, and the post-negative sign zone is 622 ms. Thus, it might be the case that the relative kinematic weakness of the negative sign zone is due to the fact that there is simply less time for the head to move (although this should be more of a factor for amplitude than for median and especially maximum velocity). To control for this, we include zone duration in the model selection process, but we cannot exclude that the relationship between zone duration and extent and its kinematics is more complex. Furthermore, while the post-negative sign zone is the longest, it is the pre-negative sign zone that is generally kinematically strongest, thus, the kinematic measures cannot be fully reduced to length. It is still interesting to observe cross-linguistic differences in the relation between the zones, as it shows that these differences are not purely mechanical.

### Computer Vision for Studying Nonmanual Markers

In this study, we have seen that computer vision (specifically, 3DDFA_v2) can be a productive and useful tool for studying formal properties of nonmanual markers in sign languages. First, it allows quantifying kinematics of these markers, which would not be possible by manual annotation alone. Second, it enables analysis of relatively large datasets, although manual annotation of the data is still required and can be a limiting factor, as in this study. The use of computer vision tools may aid in the discovery of patterns in the use of nonmanual markers, for example with regard to internal headshake kinematics, which would not be discoverable without such tools. We also expect that these tools will be useful for studying other nonmanual markers, such as head nods (as has been demonstrated by Bauer et al., 2024), eyebrow movements (Kuznetsova et al., 2021), mouth shapes, etc. We also discuss some possible future research directions using computer vision for headshake kinematics in the final section of the paper.

However, it is important to remember that, as discussed above and also in the next section, existing computer vision tools are not specifically developed for kinematic analysis of sign languages. Therefore, they need to be tested and improved, and the measurements should be interpreted with caution (see e.g., O’Brien et al., 2026). The field of quantitative analysis of nonmanual markers in sign languages and gesture is actively developing, and so the results should be treated as preliminary at this stage.

### Limitations

This study has some limitations, which can be divided into the ones related to the linguistic analysis and the ones related to the technical aspects of the computer vision tool 3DDFA_v2.

One potential limitation concerns the Russian Sign Language dataset. We acknowledge that the dataset consists of seven deaf native Russian Sign Language signers currently residing in Germany, which raises important questions regarding language contact and generalizability. In particular, prolonged residence in Germany may involve varying degrees of contact with German Sign Language, which differs from Russian Sign Language in relevant grammatical domains such as negation. Given the small sample size, it is not possible to systematically quantify or control for the extent of such contact effects across participants. We therefore explicitly restrict any typological claims based on this dataset and treat the Russian Sign Language findings as exploratory evidence rather than broadly generalizable patterns. At the same time, all participants report using Russian Sign Language in their daily communication networks, primarily with family and deaf Russian Sign Language-signing peers, and one participant reports exclusive use of Russian Sign Language with their social circle. The data were collected from individuals who migrated to Germany within the past two to five years, which reduces but does not eliminate potential long-term contact effects.

In order to compare typologically diverse sign languages to each other, we decided to annotate the data for a small number of simple and straightforward linguistic variables: headshake spreading, presence of a manual negative sign, type of the manual negative sign, and the three basic zones within headshakes (pre-negative, negative, and post-negative sign zones). The advantage of this approach is that the data were easy to annotate, as the labels apply straightforwardly to the data. The disadvantage is that these labels are in fact imprecise proxies for the more linguistically relevant features.

For the presence of a manual negative sign, as discussed, we only considered whether the manual negative sign overlapped with a headshake, not whether it occurred in the same sentence as it without overlapping. While the latter was especially common in Russian Sign Language, it still could have affected the overall results somewhat. In addition, we did not consider the syntactic position of the manual negative sign in the sentence, which can be an important factor as well. We also classified the manual negative signs into a small number of categories based on the glosses and meanings of these signs; we did not address the question whether these categories are indeed linguistically relevant for the sign languages in the dataset.

For spreading, we simply annotated whether the headshake co-occurred with a single or with multiple manual signs. A more linguistically relevant question is what the syntactic and prosodic scope of the spreading headshakes is. For example, is it the verb that is in the scope of the headshake, or does spreading also occur over the subject, adjuncts, etc.? Because we do not know enough about the syntactic structure of negative sentences for most sign languages in the dataset, it would be very challenging to carefully annotate this in a comparable way. Yet, using the very basic definition of spreading might obscure the actual patterns of spreading correlating with kinematics. Further exploration of this, possibly for individual sign languages separately, is required.

Finally, when we divided the headshake into zones, we were again driven by the necessity to have simple and cross-linguistically applicable rules. However, theories of negative headshake and its association to a manual negative sign typically operate with more syntactically and semantically informed zones. For instance, Oomen and Pfau (2017) divide headshakes into lexical, morphological and prosodic functions, and Kimmelman et al. (2024) demonstrate that in Dutch Sign Language, the related zones are kinematically different in a way compatible with this theory. These functions, however, do not map neatly onto the zones used in the current study, as a pre-negative and post-negative sign zone can be morphological (if it is the negated verb), or prosodic (if it is a different sign, not in the scope of negation), or a combination of both. It would be very interesting to study internal kinematics of headshake based on a more detailed understanding of the syntactic structure of negative sentences, probably for individual languages.

Turning to the limitations of 3DDFA_v2, previous research has shown for other tools that while they can be reliable at least for measurements of yaw in relative terms (Sargano et al., 2024), their outputs are not realistic in absolute terms—that is, the tool is not suitable for absolute measurements of rotation in degrees. In other words, a tool can often reliably distinguish between smaller and larger rotations, but it cannot provide an accurate measurement of the rotation in degrees. We have not tested this for 3DDFA_v2, so we cannot rely on the absolute estimates from this tool either. In this study, we have additionally discovered that 3DDFA_v2 rotation estimation is somewhat sensitive to the angle of the camera relative to the signer in the video. While the differences in estimations appear to be minor for reasonable angles for this specific tool, they are still not perfectly aligned, and thus minor cross-linguistic differences might not be detectable if the datasets were not recorded in very similar conditions.

In addition, the data sets are also not identical in terms of resolution of video recordings and distance between the signer and the camera. Our initial testing has shown that these parameters do not affect head rotation estimation in a meaningful way, but more systematic analysis is required.

Finally, our study is based on a small sample of only seven sign languages, which is in no way a representative sample of the world’s sign languages, and some of our sampled datasets contain quite a small set of signers. Therefore, we cannot claim our conclusions to have typological validity.

### Summary

To summarize, we have the following main findings from this exploratory study. Headshakes expressing negation vary kinematically across sign languages, in terms of duration, amplitude, and velocity. This can be interpreted as evidence of headshake grammaticalization, which leads to varying properties in different languages. This partially support Hypothesis 1.

There is also cross-linguistic variation in the role spreading has on headshake kinematics, although the general pattern is that spreading headshakes are also kinematically stronger, contrary to Hypothesis 3. We interpret this as both spreading and headshake kinematics working in tandem to make headshake more prominent in some contexts. At the same time, the presence of a manual negative sign does not appear to influence headshake kinematics directly, not even its duration, contrary to Hypotheses 2 and 4. This also aligns to some extent with the finding that, when looking at internal headshake kinematics, the headshake is often weakest over the negative sign itself. This emphasizes the independence of the headshake as a marker of negation, even in more manual dominant sign languages.

An important observation is that the kinematic properties of headshake are not directly related to the manual vs. nonmanual dominant classification of the languages in our sample, falsifying part of Hypothesis 1. We do not find that the nonmanual dominant sign languages all have a kinematically stronger headshake, or that they have the biggest difference between spreading and non-spreading headshake. Thus, the typological picture is complex: languages can be classified based on how often they use manual and nonmanual markers of negation, on the co-occurrence patterns of these markers, and on the kinematic properties of these markers, but we do not expect to find a one-to-one mapping between these features.

## CONCLUSIONS

In this paper, we analyzed kinematics of negative headshake in seven sign languages: Kata Kolok, Brazilian Sign Language, Dutch Sign Language, German Sign Language, Norwegian Sign Language, Polish Sign Language, and Russian Sign Language. We used manual annotation of headshakes for duration, and the computer vision tool 3DDFA_v2 for amplitude and velocity measurements. We considered whether headshake kinematics differ across sign languages, and whether they are affected by the presence of a manual negative sign and by spreading of the headshake across multiple signs, and signers’ age. In addition, we kinematically compared three zones within headshake: the pre-negative sign zone, the negative sign zone, and the post-negative sign zone.

For headshake duration, we found that it varies cross-linguistically, more specifically that Kata Kolok has substantially shorter headshakes than the other languages, and also that Polish Sign Language has shorter headshake than most other sign languages in our study. In addition, headshake duration is affected by spreading (as expected). We thus find evidence for cross-linguistic variation in headshake duration.

For amplitude and median and maximum velocity, we found cross-linguistic differences. German Sign Language and Brazilian Sign Language have comparatively high amplitude, while Norwegian Sign Language and Dutch Sign Language have comparatively low velocities. We found that spreading headshakes across languages have higher amplitude and median and maximum velocity than non-spreading headshakes. For amplitude, we found differences in the effect of spreading between languages: in Russian Sign Language, spreading and non-spreading headshakes have the same amplitude, unlike in the other languages. We interpret this as evidence in favor of signers using both spreading and stronger kinematics together to mark negation in a more emphatic manner, but also take it to suggest that there might be typological variation in this domain. At the same time, the presence of a manual negative sign does not significantly affect headshake kinematics. This can be interpreted as evidence that headshake is the primary marker of negation, and as such the presence of a manual negative sign is not crucial, at least for the nonmanual dominant sign languages.

For the zones within headshakes, we found a pattern that we had not predicted. We observed that, generally, the pre-negative sign headshake zone is strongest kinematically (has higher amplitude and velocity), and that the negative sign zone is mostly the kinematically weakest. This goes against the prediction from some theories according to which the headshake is lexically or morphologically linked to the manual negative sign. Importantly, we found significant interactions between zone and language affecting kinematics, which indicates that there is cross-linguistic variation in this domain.

We find no evidence of age affecting any of the kinematic measures. However, this finding is confounded by different ways age is reported in the different data sets.

Our study, while providing some exciting insights into headshake kinematics, has several limitations, which can also be used as motivation for future studies. First, since it is based on a small non-representative sample of sign languages, and on data from small numbers of signers for some of the languages, we cannot make any typological generalizations, beyond indications of where typological variability in formal properties of headshakes may lie. A larger study with more languages and signers is required. Second, we annotated the data using very crude categories in order to enable cross-linguistic comparison within a reasonable time frame. Thus, we would argue that more detailed and linguistically informed studies, possibly on single sign languages, are also required. It would be very valuable to see how different syntactic and semantic types of negation, different word orders, and different language-specific classes of negative signs affect headshake kinematics. Third, in this study, we used only a small number of kinematic measures, and only looked at them individually. An obvious next step would be to include more measures, including measures that count head turns and calculate their frequency, turn-based amplitude, etc., as in Kimmelman et al. (2024), but also using more sophisticated statistical methods and combining multiple measures.

## FUNDING INFORMATION

Funded by the European Union (ERC, NONMANUAL, project number 101039378). Views and opinions expressed are, however, those of the author(s) only and do not necessarily reflect those of the European Union or the European Research Council. Neither the European Union nor the granting authority can be held responsible for them.

## AUTHOR CONTRIBUTIONS

Vadim Kimmelman: Conceptualization; Data curation; Formal analysis; Funding acquisition; Investigation; Methodology; Project administration; Resources; Software; Supervision; Writing – original draft; Writing – review & editing. Anastasia Bauer: Conceptualization; Data curation; Investigation; Resources; Writing – original draft; Writing – review & editing. Carl Börstell: Conceptualization; Formal analysis; Investigation; Methodology; Visualization; Writing – original draft; Writing – review & editing. Jan Bulla: Formal analysis; Investigation; Methodology; Software; Writing – original draft; Writing – review & editing. Allah Bux Sargano: Investigation; Methodology; Software; Writing – original draft; Writing – review & editing. Laurence Crettenand: Data curation; Investigation; Writing – original draft; Writing – review & editing. Lorena Figueiredo: Conceptualization; Data curation; Investigation; Resources; Writing – original draft; Writing – review & editing. Anna Kuder: Conceptualization; Data curation; Investigation; Resources; Writing – original draft; Writing – review & editing. Hannah Lutzenberger: Conceptualization; Data curation; Investigation; Resources; Writing – original draft; Writing – review & editing. Dorcas Mihio: Data curation; Investigation; Software; Writing – original draft; Writing – review & editing. Marloes Oomen: Conceptualization; Data curation; Investigation; Resources; Writing – original draft; Writing – review & editing. Roland Pfau: Conceptualization; Data curation; Investigation; Resources; Writing – original draft; Writing – review & editing.

## DATA AVAILABILITY STATEMENT

The data and code for this study can be found in this Zenodo repository: https://doi.org/10.5281/zenodo.21979464.

## Notes

^1^ Following the common conventions, we gloss examples from sign languages in small caps, with nonmanual markers placed over the gloss line and aligned in scope with the manual signs. We use *hs* for headshake. Indexes in subscript that accompany pronouns and some verbs indicate spatial modification used for referential or agreement purposes. BI_1_ is a special first-person pronoun in Kata Kolok. PU stands for a palms up gesture. The glosses are translated to English, while the original glosses in the source materials are often in other written languages.^2^ Rural sign languages are sign languages that typically emerge in small isolated communities with high prevalence of deafness, and are characterized by a high number of hearing signers in the community (de Vos & Pfau, 2015).^3^ The silent articulation of a spoken language word associated with the meaning of the manual sign (see Bisnath, 2024).^4^ Vogt-Svendsen (2000) earlier reported the same pattern in an unpublished conference presentation.^5^ We should acknowledge here that for some of the sign languages in our sample, it has been argued that the headshake may fulfill a third function that is also relatively prominent, namely it may serve as a morphological affix on the verb/predicate (Oomen & Pfau, 2017; Pfau, 2002). Indeed, Kimmelman et al. (2024) provide some indication that lexical and morphological headshakes are kinematically different from prosodic headshakes in Dutch Sign Language. Because not all sign languages in our sample involve morphological headshakes, we take a different approach in the analysis presented in this paper by not dividing headshakes according to function but rather according to whether they occur on, before, or after the manual negative sign. We come back to the possible consequences of this methodological decision in the discussion. It is furthermore worth pointing out that some researchers (e.g., Neidle et al. (1999) for American Sign Language) analyze headshakes on manual negative signs as fulfilling a syntactic rather than a lexical function. This is of little consequence for our hypothesis: we consider both functions to be grammatically prominent, and therefore we expect kinematically relatively strong headshakes in both cases.^6^ Age metadata is treated differently in different datasets, sometimes with exact numeric ages, and sometimes in grouped age brackets, for which age brackets are not always equivalent across datasets. We transform the age for all the data to a numeric value; for age groups, we use the midpoint of the range (i.e., the range’s mean and median value) as a proxy. This makes the findings concerning age effects less reliable, but we do not have a better solution for these datasets.^7^ With the exception of the data from Norwegian Sign Language, where approximately a half of the conversations involved three participants, one of them being a researcher. The researcher’s production is never analyzed.^8^ We further note that currently available Russian Sign Langauge corpora from Russia contain limited amounts of spontaneous interactional data. Due to the suspension of fieldwork opportunities in Russia after 2022, data collection in Germany provided a feasible alternative for obtaining naturalistic interactional Russian Sign Language data. We therefore consider the dataset suitable for investigating interactional temporal coordination patterns, while explicitly limiting claims regarding cross-linguistic typology and generalisability.^9^ The annotators annotated the videos on a millisecond level of detail, which is more granular than the individual *frames* of the video (i.e., the highest level of visible granularity). Thus, the annotation boundaries were not originally aligned to frame boundaries, but for our inter-annotator reliability analysis, we rounded all the annotation boundaries to the nearest video frame.^10^ Our smoothing approach is potentially sensitive to the frame rate of the recording. Most of the data sets analyzed are recorded at 25 or 30 fps, with the exception of the German Sign Language dataset (50 fps). In order to ensure that this frame rate difference has not affected the results, we have downsampled the German dataset to 25 fps and compared the derived measures for the original vs. downsampled dataset. The differences turned out to be negligible. The corresponding code and results are included in the project repository.^11^ Although note that this is based on our definition of the presence of a manual negative sign, not on an average number of negative signs per negative sentence, which would be more appropriate.^12^ In a model with non-transformed duration, interaction between language and spreading is significant, and post-hoc testing shows that the effect of spreading in Kata Kolok is significantly lower than in German Sign Language and Norwegian Sign Language. However, since the residuals of the model indicate assumptions violations, we report the results of the model for log-transformed duration. See the details in the project repository.^13^ As we discussed above, Polish Sign Language cannot be characterized as nonmanual dominant, since previous literature suggests that the preferred strategy is to have a manual negative sign present. However, we think that its inclusion in this comparison is warranted here, since in our dataset, 30% of headshakes are not co-occurring with a manual negation.
